# Cost-effectiveness analysis of physical activity interventions for people with schizophrenia or bipolar disorder: systematic review

**DOI:** 10.1192/bjp.2023.52

**Published:** 2023-08

**Authors:** Huajie Jin, Oluwafunso Kolawole, Zhengwei Wang

**Affiliations:** King's Health Economics (KHE), Health Service and Population Research Department, Institute of Psychiatry, Psychology & Neuroscience, King's College London, London, UK

**Keywords:** Physical activity, cost-effectiveness, systematic review, schizophrenia, bipolar affective disorders

## Abstract

**Background:**

Clinical guidelines recommend providing physical activity interventions (PAIs) to people with schizophrenia or bipolar disorder for weight management. However, the cost-effectiveness of PAIs is unknown.

**Aims:**

To evaluate the availability and methodological quality of economic evaluations of PAIs for people with schizophrenia or bipolar disorder.

**Method:**

Four databases (MEDLINE, Embase, PsycInfo and Scopus) were searched on 5 July 2022. Based on the retrieved studies, forward and backward citation searches were conducted. Two reviewers independently selected studies for inclusion. Study quality was assessed using the Drummond checklist. Review results were presented using narrative synthesis.

**Results:**

Fourteen articles reporting nine studies were included. All included studies assessed PAIs within a multicomponent lifestyle intervention. Mixed findings were reported on the cost-effectiveness of multicomponent lifestyle intervention: three studies reported it as cost-effective; four studies reported it as not cost-effective; and two studies did not conclude whether it was cost-effective or not. Very limited evidence suggests that certain patient subgroups might be more likely to benefit from multicomponent lifestyle interventions with a PAI component: men; individuals with comorbid type 2 diabetes; and individuals who have been psychiatric hospital in-patients for ≥1 year. The quality of included studies ranged from moderate to high.

**Conclusions:**

The current economic evidence suggests that not all modalities of multicomponent lifestyle intervention including a PAI component are cost-effective for people with schizophrenia or bipolar disorder; and not all people with schizophrenia or bipolar disorder would benefit equally from the intervention. Future research is urgently needed to identify the cost-effective modality of PAI for different patient subgroups.

Obesity can reduce a person's life expectancy by up to 10 years.^1^ Compared with the general population, people with schizophrenia or bipolar disorder are at increased risk of obesity,^2^ with a 2.8- to 3.5-fold increase in those with schizophrenia and a 1.2- to 1.5-fold increase in those with bipolar disorder.^3,4^ The elevated prevalence of obesity in people with schizophrenia or bipolar disorder might be caused by antipsychotic side-effects and unhealthy lifestyle behaviours, such as lack of exercise, a poor diet and smoking.^5^ The UK is among the countries with the highest prevalence (over 40%) of obesity in people with mental illness.^6^

The efficacy of physical activity interventions (PAIs) in reducing the risk of obesity is well established in the literature.^7^ The clinical guidelines developed by the National Institute for Health and Care Excellence (NICE) in the UK recommend that people with schizophrenia or bipolar disorder, especially those taking antipsychotics, should be offered a combined healthy eating and physical activity programme by their mental healthcare provider.^8,9^ Similarly, the World Health Organization (WHO) guidelines recommend that behavioural lifestyle interventions including physical activity and a healthy diet should be considered for all people with severe mental illness (SMI) who are overweight or obese or at risk of becoming overweight or obese.^10^

It should be noted that the positive recommendations of PAIs made by NICE and WHO were based on clinical effectiveness evidence alone, without any economic evidence, as the systematic reviews conducted by the NICE guideline development team did not identify any economic evidence for PAIs for people with schizophrenia or bipolar disorder, and the review conducted by the WHO did not search for economic evidence. Since the publication of the NICE guidelines, Verhaeghe et al^11^ conducted a systematic review of economic evidence for PAI in people with SMI, but they did not find any evidence either. This might because first, Verhaeghe et al's literature search was conducted in 2010; and second, their review was limited to trial-based economic evaluations and ignored other types of economic evaluation, such as those based on economic models. Economic evaluations can be based on clinical trials, decision analytical modelling, cohort studies or database studies. Of these four analytical frameworks, trial-based studies and decision analytical modelling are more commonly used in healthcare economic evaluation. In a trial-based economic evaluation, the economic evaluation is carried out alongside a clinical trial, with the trial providing the main source of input data (e.g. baseline event rates, treatment effects, adverse event rates, resource use and utility values, as appropriate). In a decision analytical model (often termed a model for short), the economic evaluation is carried out within a mathematical framework imitating the current and proposed system of care, with input data obtained from multiple sources. Since 2010, several cost-effectiveness studies of PAIs have been published for people with schizophrenia or bipolar disorder, including both trial-based and model-based analyses.^12,13^ Therefore, a new systematic review on this topic is warranted.

To fill the evidence gap, our review aims to evaluate the availability and methodological quality of economic evaluations of any PAI for weight management in people with schizophrenia or bipolar disorder. Specific objectives were as follows:

- to identify economic evaluations of PAIs as a weight management intervention in people with schizophrenia or bipolar disorder
- to critically examine the methodological quality and the risk of bias of existing health economic evaluations
- to summarise the conclusions reported by existing health economic evaluations.

## Method

This systematic review was conducted in line with the Preferred Reporting Items for Systematic Reviews and Meta-Analyses (PRISMA) recommendations for reporting systematic reviews and meta-analyses of studies that evaluate healthcare interventions.^14^ The PRISMA checklist can be found in the Section 1 of the Supplementary Materials available at https://doi.org/10.1192/bjp.2023.52. The protocol for the systematic review was registered on PROSPERO, the National Institute of Health Research Database (registration: CRD42022359492).

### Inclusion and exclusion criteria

The inclusion and exclusion criteria were defined *a priori*. Studies were included if they met all the following criteria: (a) studies reporting full or partial health economic evaluations based on any analytical frameworks (a full economic evaluation is defined as ‘the comparative analysis of alternative courses of action in terms of both their costs and consequences’, whereas a partial economic evaluation focuses solely on costs^15^); (b) studies focusing on patients of any age who have been diagnosed with schizophrenia or schizophrenia-like illnesses or bipolar disorder using any criteria; and (c) studies assessing PAIs or exercise interventions for weight management. Such interventions could be used alone or in conjunction with other interventions (e.g. as part of a multicomponent lifestyle intervention). When used with other interventions, the PAI or exercise should be the main or active element of the intervention. Studies were excluded if they met any of the following criteria: (a) articles in a foreign language with no English version of the full text; (b) protocols, reviews, commentaries, letters, editorials or studies without abstract and full text.

### Search strategy

On 5 July 2022, a thorough and systematic search was conducted on the following electronic databases: MEDLINE (from 1946 to 5 July 2022), Embase (from 1974 to 5 July 2022), PsycInfo (from 1806 to Week 4, June 2022) and Scopus (from 1976 to 5 July 2022). In addition, backward citation searches were conducted on the reference list of included studies and relevant systematic reviews. Forward citation searches were conducted on Scopus and Google Scholar. A search on PROSPERO (www.crd.york.ac.uk/prospero), an electronic register of prospectively registered systematic reviews, shows that there were no ongoing systematic reviews on the economic evaluation of PAIs as a weight management intervention in people with schizophrenia or bipolar disorder. The search strategies and results can be found in the Supplementary Materials, Section 2.

### Study selection

Two reviewers (O.K. and Z.W.) independently reviewed the titles and abstracts of all the retrieved articles to determine the potential eligibility of studies for this review. All retrieved articles were imported to Rayyan, a screening web tool system, where duplicates were removed (www.rayyan.ai, accessed on 4 November 2022).^16^ Rayyan was also used for the first-round screening (based on title and abstract) and second-round screening (based on full text) of the retrieved articles. The reviewers were masked to each other's screening. Any disagreements between the two reviewers were resolved by discussion with a third reviewer (H.J.). Cohen's kappa score was calculated to determine the inter-rater reliability.

### Data extraction

The data extraction tables used for this review were adapted from a previous systematic review of economic evaluation^17^ and were piloted for three randomly selected studies before being used in this review. The following information was extracted from the included studies: author and year, country, setting, intervention, comparator, type of economic evaluation, time horizon, perspective, study population, consideration of patient subgroups (e.g. potential subgroups considered, and the rationale for conducting or not conducting subgroup analysis), modelling method (if applicable), conflict of interest, cost-effectiveness results, results of sensitivity analyses, conclusions reported by the author and any information relevant to the quality assessment criteria. The cost-effectiveness results include cost outcomes, clinical outcomes (e.g. quality-adjusted life years (QALYs), body mass index (BMI) and waist circumference) and incremental cost-effectiveness ratios (ICERs), if applicable.

### Quality assessment

The Drummond checklist has been widely used to assess the risk of bias and quality of evidence for both trial-based and model-based economic evaluations.^15^ The checklist has two versions: a short 10-point version consisting of 10 questions and a full 35-point version consisting of 35 questions. Each question can be answered with ‘yes’ (if the paper fully meets the criterion), ‘no’ (if the paper does not meet the criterion), ‘can't tell answers’ (if it is unclear whether the paper meets the criterion or not), ‘partly’ (if the paper partially meets the criterion) or ‘N/A’ (if not applicable). Both versions were used for the quality assessment in this review.

The quality assessment was conducted by one reviewer (O.K.). The results of quality assessment of three randomly selected included studies were checked by another reviewer (Z.W.). Any disagreements between reviewers were resolved by discussion with a third reviewer (H.J.).

### Method of synthesis

Meta-analysis was not deemed to be appropriate for analysing the results of cost-effectiveness analysis, as the results of single economic studies may not be transferable between different places and time owing to differences in local economic situations and healthcare systems and widespread methodological heterogeneity.^18^ Therefore, the data extracted for this review were synthesised using the narrative synthesis guideline developed by Popay and colleagues.^19^

The certainty of evidence of the trial-based studies was assessed following the Grading of Recommendations Assessment, Development and Evaluation (GRADE) approach, using the GRADEpro GDT tool (online version, accessed 18/11/2022 at gradepro.org). The certainty of the evidence was categorised as high, moderate, low or very low. The GRADE approach was not applied to model-based studies as the relevant guidance is still under development and has not yet been published.^20^

## Results

### Study selection

**Fig. 1:**
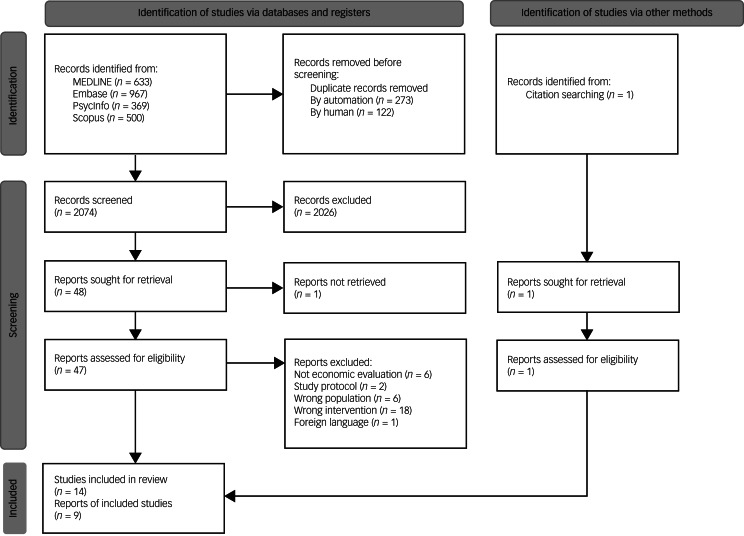
PRISMA flowchart for the literature search.

Figure 1 shows the PRISMA flowchart for the included studies. A total of 2469 articles were retrieved from the electronic searches. One additional study which meets the inclusion criteria was identified on Google Scholar through backward citation searching.^12^ This article was not retrieved through the electronic searches because the article was not indexed in any of the mainstream databases searched. Therefore, in total, 2470 articles were identified for screening. After removing 395 duplicates, 2074 articles remained. Of these, 2026 articles were excluded at the first round of screening. The full texts of the remaining 48 articles were ordered. Of the 47 articles whose full texts are available, 33 studies were excluded. Reasons for exclusion include not assessing the intervention of interest (18/33, 54.5%) or the population of interest (6/33, 18.2%),^21–26^ not an economic evaluation (6/33, 18.2%),^27–32^ protocols (2/33, 6.1%)^33,34^ and not in English (1/33, 3.0%).^35^ The authors of the two protocols were contacted to ask for the results of their study but no responses were received. In total, 14 articles reporting 9 studies met the predefined inclusion criteria and were included in the review. The Cohen's kappa score for inter-rater agreement was 0.49, implying a moderate agreement between the investigators.^36^ A list of included studies is reported in the Supplementary Materials, Section 3.

### Study description

Table 1 summarises the characteristics of the nine included studies. All included studies were conducted in high-income countries: the UK (4/9, 44.4%),^12,13,37,38^ the USA (2/9, 22.2%),^39,40^ The Netherlands (2/9, 22.2%)^41,42^ and Belgium (1/9, 11.1%).^43^ The settings of the interventions were community mental health services (5/9, 55.6%),^37–41^ primary care (2/9, 22.2%),^12,13^ a sheltered housing organisation (1/9, 11.1%)^43^ and in-patient care (1/9, 11.1%).^42^

**Table 1:** Study characteristics

Author and year	Country	Setting	Intervention	Comparator	Type of economic evaluation	Time horizon	Analytical framework	Perspective	Study population	Conflict of interest
Deenik et al 2022^42^	The Netherlands	In-patient hospital	Multidisciplinary lifestyle-enhancing treatment tailored to individual patients' needs. The active programme part of this intervention consisted of sports-related activities (e.g. walking, running, yoga, biking, indoor team sports), work-related activities (e.g. gardening and working in services within the hospital), psychoeducation (e.g. about medication side-effects, dietary habits) and daily living skills training (e.g. shopping, cooking)	Patients who received TAU continued their treatment in their wards led by their own (non-participating) head practitioner, which mainly concerned pharmacological treatment and a less-structured day programme that did not include any supported lifestyle interventions or adjustments	CUA	18 months	Cohort study (*n* = 114)	Healthcare	Patients with schizophrenia spectrum and other psychotic disorders who had been in a psychiatric hospital for at least 1 year	None
Heslin et al 2017^38^	UK	Community mental health service	Multicomponent lifestyle intervention targeting exercise, diet, tobacco smoking, alcohol use, cannabis use, other illegal substances and diabetes (where applicable) + CBT + TAU	TAU – care coordinators were given training on physical health awareness + health promotion leaflets	CUA	15 months	Multicentre, two-arm, parallel cluster RCT (*n* = 406)	Healthcare and social care and societal perspectives	People with psychotic disorder (ICD-10 diagnosis: F20-29, F31.2, F31.5) aged 18–65 years under the care of a community mental health team registered on an enhanced level of the Care Approach Programme (CPA) or equivalent)	None
Holt et al 2019^37^	UK	Community mental health service	Four 2.5 h weekly group sessions on psychoeducation and behavioural change targeting diet and physical activity followed by 1:1 support contact, mostly by telephone, every 2 weeks for the remainder of the intervention period; further 2.5 h group-based booster sessions at 4, 7 and 10 months + TAU	TAU – printed advice on lifestyle and the risks associated with weight gain for all participants	CUA	12 months	Two-arm, parallel group RCT (*n* = 412)	Healthcare and social care and societal perspective	Adults (≥18 years) with schizophrenia, schizoaffective disorder or first-episode psychosis prescribed an antipsychotic for ≥1 month and able and willing to participate in a group education programme. Participants had a BMI ≥25 kg/m^2^ (≥23 kg/m^2^ for South Asian and Chinese backgrounds) or expressed concern about their weight	None
Janssen et al 2017^40^	USA	Out-patient community mental health service	Group weight-management sessions, three times a month; individual weight-management sessions monthly; group exercise sessions 3 days per week involving moderate-intensity aerobic exercise; exercise classes were led by a trained member of the study staff	Standard nutrition and physical activity information at baseline. Health classes were offered quarterly, with content unrelated to weight (e.g. cancer screening)	CEA	18 months	RCT (*n* = 291)	Healthcare	Adults with overweight or obesity from community psychiatric rehabilitation out-patient programmes; 58.1% had schizophrenia or a schizoaffective disorder, 22.0% had bipolar disorder, and 12.0% had major depression	None
Looijmans et al 2020^41^	The Netherlands	Community mental health centres	Months 1–6: patients’ lifestyle behaviour was screened by nurses during regular care visits once every 2 weeks, using a web tool ‘Traffic Light Method’. Nurses created a lifestyle plan with specific goals with the patients. The intervention addressed diet and physical exercise and incorporated behavioural techniques. Months 7–12: further lifestyle plans were mapped out between patients and nurses. Plans were evaluated until the trial ended	Usual care – medical problems were tackled immediately according to protocol, whereas lifestyle guidance was provided on request of the patients	CUA	12 months	Clustered RCT (*n* = 244)	Societal and third-party payer^a^	Patients managed by community mental health centres, whose annual physical screening showed one or more increased metabolic risk factors: waist circumference >88 cm (females) or 102 (males), BMI > 25 kg/m^2^, fasting glucose levels >5.6 mmol/L, or HbA_1c_ > 5.7% or >39 mmol/mol. Diagnoses include psychotic disorder (75%), bipolar disorder (15%), complex personality disorders (10%)	None
Meenan et al 2016^39^	USA	Community mental health centres	Months 1–6: weekly group meetings covering topics on nutrition, physical activity (mainly walking); months 7–12: monthly group meetings to support the maintenance of behaviour change and weight loss + individual monthly contacts with intervention group facilitators; plus 12 months follow-up	Usual care – not described, but participants in this group had no obligation to the study and were free to initiate any weight-loss effort on their own	CUA	24 months	Parallel-arm, multi-site RCT (*n* = 200)	Healthcare and societal	Individuals ≥18 years of age who were taking antipsychotic medications for at least 30 days prior to enrolment, and who had a BMI > 27. Diagnoses include affective psychosis (38%), bipolar disorder (32%), schizophrenia spectrum disorders (29%) and PTSD (2%)	None
Osborn et al 2018^13^	UK	GP practice – Primary care	Multicomponent lifestyle intervention to improve diet or physical activity levels, reduce alcohol, and quit smoking; participants were offered weekly to fortnightly appointments for up to 6 months	British Heart Foundation leaflets were mailed to participants. The usual clinical pathways for cardiovascular disease risk factors were continued in this group	CUA	12 months	Clustered RCT (*n* = 327)	Healthcare	Participants aged 30–75 years with severe mental illness (schizophrenia, bipolar disorder or psychosis), who had raised cholesterol concentrations (≥5.0 mmol/L) or a total: HDL cholesterol ratio of 4.0 mmol/L or more and one or more modifiable cardiovascular disease risk factors. About half of the population had bipolar disorder, almost one-third had schizophrenia or schizoaffective disorder and almost one-fifth had other psychoses	None
Park 2014^12^	UK	Primary care	Group-based lifestyle intervention including psychosocial education on diabetes, healthy eating habits and physical activity. Self-monitoring: participants could record their level of exercise daily	Usual care – two general practitioner visits + dietitian referral when necessary	CUA	6 months	Model (decision tree)	Healthcare	Middle-aged adults with chronic long-term schizophrenia and comorbid type 2 diabetes	None
Verhaeghe et al 2014^43^	Belgium	Sheltered housing organisation	Ten psychoeducational and behavioural group-based sessions in a 10-week period; group-based exercise in the same 10-week period (weekly 30 min supervised walking sessions); and individual support from the mental health nurses during the 10-week intervention. Intervention included TAU	TAU – weekly meetings between the mental health nurse and the resident to discuss topics such as how to cope with the psychiatric illness, somatic health, household tasks and financial issues	CUA	20 years	Clustered RCT (*n* = 284) + Markov model	Healthcare	Individuals with mental disorder living in sheltered housing. Diagnoses: schizophrenia (37.9%), mood disorder (24.5%), substance misuse (15.9%), personality disorder (14.4%) and other (7.2%)	None

BMI, body mass index; CBT, cognitive–behavioural therapy; CEA, cost-effectiveness analysis; CUA, cost–utility analysis; HbA_1c_, glycated haemoglobin; PTSD, post-traumatic stress disorder; RCT, randomised controlled trial; TAU, treatment as usual.

a. Looijmans et al used one costing perspective (societal) for their economic evaluation, and two costing perspectives for their budget impact analysis (societal and third-party payer).

Regarding the population, all included studies recruited people with a mixed diagnosis of mental disorders except Park,^12^ which recruited only people with schizophrenia and comorbid type 2 diabetes mellitus. People with schizophrenia were included in all nine studies, whereas people with bipolar disorder were included in six studies.^13,38–41,43^ Regarding the intervention assessed, all included studies compared the cost-effectiveness of usual care alone with MBLIs (that included a PAI component) with or without usual care. Two studies (2/9, 22.2%)^37,38^ compared the intervention plus usual care with usual care only. Five studies assessed group-based interventions (5/9, 55.6%) and the other four assessed individual-based interventions (4/9, 44.4). The PAI component within the multicomponent lifestyle intervention varied greatly, including group or individual aerobic exercises, low to medium intensity exercises, walking, running, yoga, biking and indoor team sport to achieve and maintain weight loss. Various time horizons were used by the included studies: 6 months (1/9, 11.1%),^12^ 12 months (3/9, 33.3%),^13,37,41^ 15 months (1/9, 11.1%),^38^ 18 months (2/9, 22.2%),^40,42^ 24 months (1/9, 11.1%)^39^ and 20 years (1/9, 11.1%).^43^

The types of economic evaluation adopted by included studies were cost–utility analysis (CUA) (8/9, 88.9%), and cost-effectiveness analysis (CEA) excluding CUA (1/9, 11.1%).^40^ CEA is one of the most commonly used types of economic evaluation in healthcare. Within a CEA, effectiveness outcomes are valued using a single disease-specific measure. For example, Janssen et al^40^ – the only included study that adopted a CEA excluding CUA framework – used per kilogram loss of weight as the single disease-specific measure. CUA is generally considered to be a special case of CEA; the two analyses differ in how outcomes are measured, but the analytical approach is the same and the results are presented in the same way. Within a CUA, effectiveness is measured in terms of generic ‘healthy years’. Of the eight CUA studies included, all used QALYs as the effectiveness outcome. Seven studies (7/9, 77.8%) were pure trial-based economic evaluations, one study was a pure model-based economic evaluation^12^ and one study was based on both trial and model.^43^ In terms of costing perspective, five studies used a healthcare perspective only (5/9, 55.6%); three studies (3/9, 33.3%) used two perspectives: a healthcare and social care perspective and a societal perspective; ^37–39^ and one study used one perspective (societal) for their cost-effectiveness and two perspectives for their budget impact analysis (societal and third-party payer).^41^ Two studies reported that they had conducted subgroup analysis,^37,43^ but only one of them reported results of subgroup analysis.^43^ None of the included studies reported on their consideration of patient subgroups, such as potential subgroups considered, the rationale for conducting or not conducting subgroup analysis, or any limitations.

### Quality assessment

For the sake of simplicity, only the results of the 10-point version of the Drummond checklist are summarised here. The results of the full 35-point version are reported in the Supplementary Material, Section 4.

**Fig. 2:**
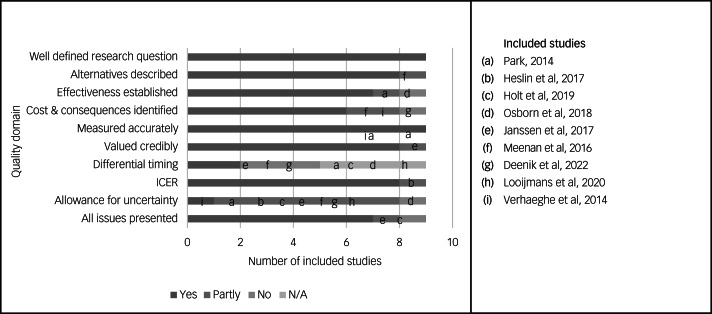
Quality assessment of included studies. 0049CER, incremental cost-effectiveness ratio; N/A, not applicable.

The results of the 10-point Drummond checklist are reported in Table 2 and graphically represented in Fig. 2. None of the included studies met all ten criteria outlined in the checklist. Criteria where studies performed poorly were the use of a time horizon not long enough to reflect all important differences in costs and outcomes (8/9, 88.9%), failure to test all important parameters whose values were uncertain in sensitivity analysis (8/9, 88.9%), failure to discount costs or consequences for differential timing despite adopting a time horizon of over 1 year (5/9, 55.5%), failure to capture all important costs or consequences for each intervention under assessment (3/9, 33.3%) and not performing an incremental analysis of costs and consequences of alternatives (1/9, 11.1%).

**Table 2:** Summary of study results

Author and year	Incremental cost (control versus intervention)	Incremental effectiveness (control versus intervention)	Incremental cost-effectiveness ratio (ICER) (control versus intervention)	Conclusion
Deenik et al 2022^42^	Incremental healthcare cost: –€736.28 (95% CI –2145.18 to 672.62)	Incremental QALY with outliers: 0.06 (95% CI –0.08 to 0.20) Incremental QALY without outliers: 0.06 (95% CI –0.08 to 0.20)	Results of deterministic analysis showed that intervention dominated control Results of PSA showed that ≥80% of the repetitions delivered health improvements with the intervention compared with control, and on all outcomes the majority of repetitions demonstrated that intervention resulted in more health improvements and fewer costs compared with control (36% in weight to 57% in systolic blood pressure)	Intervention did not increase healthcare costs while improving health outcomes
Heslin et al 2017^38^	Incremental cost from the healthcare perspective: £95 (95% CI –£1410 to £1599) Incremental cost from the societal perspective: £675 (95% CI –£1039 to £2388)	Incremental QALYs estimated from the SF-36: −0.00 (95% CI −0.01 to 0.00) Incremental QALYs estimated from the EQ-5D-3 L: 0.00 (95% CI −0.01 to 0.02)	Because the difference in QALYs between intervention and control group was almost zero, it was not useful to calculate a deterministic ICER. Results of PSA showed that the probability of the intervention being cost-effective did not exceed 0.4 for any of the examined WTP thresholds for QALY gains. Results are robust to all sensitivity analyses conducted.	No evidence of cost-effectiveness for intervention
Holt et al 2019^37^	Incremental cost from the healthcare and social care perspective: £802 Incremental cost from the societal perspective: £1027	0.0035 QALYs	ICER from the healthcare perspective: £246 921 per QALY gained ICER from the societal perspective: £367 543 per QALY gained	The intervention was not cost-effective over the 12-month intervention period
Janssen et al 2017^40^	Healthcare cost: $95 per participant per month	Weight loss for the intervention group: 3.4 kg	$501 per kilogram lost. In univariate sensitivity analysis, costs ranged from $402 to $725 per kilogram lost. Results are sensitive to the number of staff members per contact session, average time per session and cost of supplies	A WTP threshold was not reported; therefore, no conclusion can be drawn regarding the cost-effectiveness of the intervention
Looijmans et al 2020^41^	Incremental cost from the societal perspective: €2516 (95% CI −€12 592 to €17 423)	Incremental QALY: −0.001 (95% CI −0.033 to 0.032).	Because the difference in QALYs between intervention and control group was almost zero, it was not useful to calculate a deterministic ICER. Results of PSA showed that the probability of the intervention being cost-effective with regard to QALYs was around 40% for the whole range of thresholds explored. Results are robust to all sensitivity analysis conducted	The intervention was not cost-effective at 12 months
Meenan et al 2016^39^	Incremental cost from the healthcare perspective: ranging from $4365 to $5687. Incremental cost from the societal perspective: $5493	Utility gain was too small, hence QALY was not calculated. Participants in the intervention group lost an average of 4.4 kg by the end of the 6-month intensive intervention period and maintained a 2.6 kg loss at 12 months	ICER ranged from $1940 to $2527 per kilogram lost, depending on which costs and which patient group were included (completers versus intention to treat). Results are sensitive to overheads rate and staff salary	A WTP threshold was not reported; therefore, no conclusion can be drawn regarding the cost-effectiveness of the intervention
Osborn et al 2018^13^	Incremental healthcare cost: –£895 (95% CI −1631 to −160; *P* = 0.012)	Incremental QALYs: −0.011 (95% CI −0.034 to 0.011; *P* = 0.41)	Deterministic ICER for control: £81 363 per QALY Results of PSA showed that at a £20 000 WTP for a QALY gained, the probability for the intervention to be cost-effective was 89%	The intervention was potentially cost-effective
Park 2014^12^	Incremental healthcare cost: £10.76	Incremental QALY: 0.12	ICER = £676.89 per QALY gained. Results of PSA showed at a WTP of £20 000 per QALY gained, there was a 52.8% probability of the intervention being cost-effective. Results are sensitive to the cost of the intervention	The intervention was cost-effective in the short term
Verhaeghe et al 2014^43^	Incremental healthcare cost for men: €228 Incremental healthcare cost for women: €263	Incremental QALY for men: 0.008 (95% CI 0.003–0.014) Incremental QALY for women: 0.007 (95% CI 0.002–0.011).	ICER for men: €27 096 per QALY ICER for women: €40 139 per QALY Scenario analysis assuming an increase in quality of life due to BMI decrease resulted in much better cost-effectiveness in both men (€3357 per QALY) and women (€3766 per QALY). Results are sensitive to the intervention effect and the intervention cost	The intervention was cost-effective in men but not in women

BMI, body mass index; EQ-5D-3 L, 3-level five-dimensional EuroQoL; PSA, probabilistic sensitivity analysis; QALY, quality-adjusted life year; SF-36, 36 item Short-Form Health Survey; WTP, willingness to pay.

### Findings of included studies

The main findings reported by the included studies are summarised in Table 2. In summary, compared with usual care:

- five studies found that PAI was more effective and more expensive;^12,39–41,43^ for these five studies, whether PAI was considered to be cost-effective or not depended on the local willingness-to-pay (WTP) threshold for an additional unit of effectiveness (e.g. QALY);
- one study found that PAI was more effective and equally expensive, and therefore was cost-effective;^42^
- one study found that PAI was equally effective and equally expensive, and therefore as cost-effective as usual care;^38^
- one study found that PAI was equally effective and more expensive, and therefore not cost-effective;^37^
- one study found that PAI was less effective and less expensive.^13^

The details of each of the above studies are as follows.

### PAI more effective and more expensive than usual care (*n* = 5 studies)

Janssen et al conducted a trial-based economic evaluation to compare the cost-effectiveness of an 18-month group intervention including group exercise sessions and moderate-intensity aerobic exercise with no intervention in the USA.^40^ This economic evaluation was based on a randomised controlled trial (RCT) which recruited 291 participants with SMI and overweight or obesity from community psychiatric rehabilitation out-patient programmes;^44^ 58.1% of participants had schizophrenia or a schizoaffective disorder, 22.0% had bipolar disorder and 12.0% had major depression. They found that participants in the intervention group lost, on average, 3.2 kg more than the control group over 18 months. The ICER of the intervention was $501 per kilogram lost, which was higher than many commercial weight-loss plans or lifestyle interventions. One-way sensitivity analyses (which can be used to assess the sensitivity of the results to variations in a specific input parameter or assumption) were conducted for a series of parameters related to the cost of providing the intervention. The results of sensitivity analyses showed that varying the number of staff members per contact session changed the overall cost the most. Probabilistic or bootstrapping sensitivity analysis (which can be used to quantify the level of confidence in the results, in relation to the joint uncertainty of multiple parameters simultaneously) was not conducted. The findings of Janssen et al^40^ are similar to the findings of the trial-based economic evaluation conducted by Meenan et al in the USA, who reported that participants with SMI lost an average of 4.4 kg at the end of the 6-month intensive group-based lifestyle intervention period and maintained a 2.6 kg loss at 12 months.^39^ The economic evaluation of Meenan et al was based on an RCT which recruited 200 individuals 18 years or older who had been taking antipsychotic medications for at least 30 days prior to enrolment and who had a BMI ≥27.^45^ The recruited participants had a mixed diagnosis of SMI, including affective psychosis (38%), bipolar disorder (32%), schizophrenia spectrum disorders (29%) and post-traumatic stress disorder (PTSD) (2%). Meenan et al reported that the ICER of the intervention ranged from $1940 to $2527 per kilogram lost depending on which costs and which patient group were included (e.g. completers versus intention to treat). One-way sensitivity analyses were conducted for certain measures and assumptions, the results of which showed that the single largest individual effects on estimated ICERs resulted from lowering the overheads rate and reducing salaries by 25%. Probabilistic or bootstrapping sensitivity analysis was not conducted. Neither Janssen et al^40^ nor Meenan et al^45^ reported a WTP threshold; therefore, no conclusion can be drawn regarding the cost-effectiveness of the interventions. Verhaeghe et al used a combination of trial and Markov models to compare the 20-year cost-effectiveness of group-based exercise plus usual care with usual care alone for people with SMI in Belgium.^43^ The within-trial analysis was based on a cluster preference RCT which recruited 284 individuals with SMI living in sheltered housing.^46^ The diagnoses of the recruited participants included schizophrenia (37.9%), mood disorder (24.5%), substance misuse (15.9%), personality disorder (14.4%) and other (7.2%). An age- and gender-dependent Markov model was then used to project the 20-year cost-effectiveness of the intervention, assuming a repeated yearly implementation of the intervention. Based on the results of deterministic analysis, they reported that the 20-year ICER of the intervention was €27 096 per QALY for men and €40 139 per QALY for women, results that are very similar to the average ICER based on the probabilistic sensitivity analysis (PSA) results (€27 624 per QALY for men and €36 571 per QALY for women). At a cost-effectiveness threshold of €30 000 per QALY, the intervention was deemed to be cost-effective for men but not for women. Comprehensive one-way sensitivity analyses were conducted to test a range of parameters/assumptions; the results showed that the model is most sensitive to the intervention effect and intervention cost in both men and women. The probability of the intervention being cost-effective was not reported.

Looijmans et al in The Netherlands conducted an economic evaluation and budget impact analysis for a web tool that can be used to generate a personalised lifestyle plan (including physical exercise) for people with SMI and monitor their progress.^41^ Their analysis was based on a pragmatic RCT which recruited 244 people with SMI managed by community mental health centres, whose annual physical screening showed one or more increased metabolic risk factors: waist circumference >88 cm (females) or >102 cm (males), BMI > 25 kg/m^2^, fasting glucose levels >5.6 mmol/L or glycated haemoglobin (HbA_1c_) >5.7% or >39 mmol/mol.^47^ They found that the intervention did not result in higher QALYs but reduced participants’ waist circumference by −1.03 cm (95% CI −3.42 to 1.35 cm; non-significant) after 12 months. The intervention was not considered to be cost-effective at 12 months. The results were robust for the number of participants per trained coach and the use of complete data only (one-way/scenario sensitivity analysis was only conducted for these two changes). The results of probabilistic sensitivity analysis showed that the probability of the intervention being cost-effective in terms of QALYs is around 40% for the whole range of thresholds explored (€0–300 000 per QALY). Park conducted a model-based economic evaluation using a decision tree for middle-aged adults with chronic schizophrenia and comorbid type 2 diabetes in primary care in the UK.^12^ The intervention assessed was a group-based lifestyle intervention that focused on psychosocial education on diabetes, healthy eating habits and physical activity. Park found that the intervention resulted in reduced BMI (from 33.6 to 32.9 kg/m^2^, *P* < 0.001) after 6 months.^12^ The 6-month ICER of the intervention was £81.23 per extra unit of BMI loss and less than £700 per QALY gained. One-way and two-way sensitivity analyses were conducted for several parameters/assumptions related to the cost of the intervention and usual care; the results showed that when the cost of the intervention increased from £173 to £399, the intervention was no longer cost-effective. The results of PSA showed that the intervention had a 52.8% probability of being cost-effective, at a WTP of £20 000 per QALY gained. Park concluded that the intervention was cost-effective in the short term, but refresher sessions might help prolong the beneficial effects accrued beyond 6 months.

### PAI more effective and equally expensive compared with usual care (*n* = 1 study)

Deenik et al assessed the cost-effectiveness of a holistic lifestyle intervention tailored to individual patients with SMI in a psychiatric hospital in The Netherlands.^42^ The intervention focused on decreasing sedentary behaviour, increasing physical activity and improving dietary habits. Resource use data before and after the implementation of the intervention were retrospectively retrieved from an 18-month cohort study.^48^ In total, 114 individuals with schizophrenia spectrum and other psychotic disorders, who had been in-patients for at least 1 year were recruited. The results of adjusted linear regression models (adjusted for baseline costs and baseline differences) showed a non-significant decrease of €736.28 per patient per quarter year (95% CI –2145.18 to 672.62) in favour of the intervention. The results of PSA showed statistically non-significant cost savings against health improvements for all health-related outcomes (e.g. weight, blood pressure and quality of life) in the intervention group compared with usual care. Deenik et al concluded that PAI did not increase healthcare costs while improving health outcomes.

### PAI equally effective and equally expensive compared with usual care (*n* = 1 study)

Heslin et al conducted a trial-based economic evaluation in the UK to compare the cost-effectiveness of a 15-month integrated health promotion intervention (IMPaCT) plus treatment as usual (standard community mental health team care) with treatment as usual alone in people with established psychosis.^38^ IMPaCT aimed to improve health and reduce substance use. It targeted exercise, diet, tobacco smoking, alcohol use, cannabis use, other illegal substances and diabetes (where applicable) and included cognitive–behavioural therapy (CBT) and usual care. The economic evaluation was based on a pragmatic multi-centre phase III cluster RCT which recruited 406 participants with psychotic disorder (ICD-10 diagnosis: F20–29, F31.2, F31.5), who were 18–65 years old and under the care of community mental health teams. They found that IMPaCT showed no difference in cost or health benefit compared with usual care. Results of bootstrapping showed that the probability of the health promotion intervention being cost-effective did not exceed 0.4 for WTP thresholds ranging from £0 to £50 000 per QALY. Comprehensive determinist sensitivity analyses were conducted and the conclusion was robust for all parameters/assumptions tested.

### PAI was equally effective and more expensive compared with usual care (*n* = 1 study)

Holt et al conducted a trial-based economic evaluation in the UK to compare the cost-effectiveness of usual care with a 12-month intervention (weekly group sessions on psychoeducation and behavioural change targeting diet and physical activity).^37^ This economic evaluation was based on an RCT that recruited 412 patients with schizophrenia, schizoaffective disorder or first-episode psychosis, who had been prescribed an antipsychotic for ≥1 month, were able and willing to participate in a group education programme and had a BMI ≥25 kg/m^2^ (≥23 kg/m^2^ for South Asian and Chinese backgrounds) or had expressed concern about their weight. They found that compared with usual care, the intervention was more expensive but did not show improvement in QALY or weight loss over the 12-month intervention period. A deterministic sensitivity analysis was conducted to test missing data mechanisms and a subgroup analysis was conducted for participants with different duration of SMI, but the results of these analyses were not reported. PSA was not conducted.

### PAI was less effective and less expensive than usual care (*n* = 1 study)

Osborn et al conducted a trial-based economic evaluation in the UK to compare the cost-effectiveness of usual care with a primary care intervention that aimed to decrease total cholesterol concentrations and cardiovascular disease risk for people with SMI (bipolar disorder, schizophrenia or schizoaffective disorder and other psychoses).^13^ This economic evaluation was based on an RCT that recruited 327 participants aged 30–75 years with SMI, who had raised cholesterol concentrations (≥5.0 mmol/L) or a total HDL cholesterol ratio of ≥4.0 mmol/L and one or more modifiable cardiovascular disease risk factors. They reported that at 12 months, compared with usual care, the intervention was associated with a lower cost (mean difference –£895, 95% CI –1631 to –160) and lower QALY gains (−0.011, 95% CI −0.034 to 0.011). The results of bootstrapping showed that the probability of the intervention being cost-effective was 98% at a £0 per QALY WTP threshold and 89% at a £20 000 per QALY WTP threshold. No results of deterministic sensitivity analyses were reported.

### GRADE evaluation

The GRADE table for the eight trial-based economic evaluations are reported in the Supplementary Materials, Section 5. All eight studies were rated as moderate except Deenik et al.^42^ Deenik et al was rated as low because it was based on an observational study.

## Discussion

### Summary of the main findings

Our review identified 14 papers reporting 9 original studies about the cost-effectiveness of MBLIs including a PAI component for people with schizophrenia or bipolar disorder. All included studies were published after 2013 and conducted in high-income countries (UK, USA, The Netherlands and Belgium).

None of the included studies assessed the cost-effectiveness of PAI alone; instead, they all assessed PAI as a component of MBLIs. Mixed results about the cost-effectiveness of MBLIs including a PAI component were reported. In summary, three studies reported the intervention to be cost-effective;^12,13,42^ two studies reported the intervention to be not cost-effective;^37,41^ one study reported the intervention to be cost-effective for men but not for women;^43^ one study reported the intervention to be as cost-effective as usual care;^38^ two studies reported the intervention to be more effective and more expensive than usual care, but they did not set a WTP threshold.^39,40^ Therefore, it was not possible to conclude whether the intervention was cost-effective or not.

This finding of our review (i.e. mixed conclusions reported by different studies) is similar to the findings of previous systematic reviews of economic evaluations of other interventions for people with schizophrenia or bipolar disorder. For example, a recent systematic review conducted by Jin et al identified 60 model-based analyses assessing the cost-effectiveness of antipsychotic medications for people with schizophrenia.^49,50^ However, Jin et al reported that it was not possible to identify the most cost-effective antipsychotic for schizophrenia as inconsistent conclusions have been reported by different studies. A systematic review conducted by Shields et al identified 12 trial-based economic evaluations of psychological therapies for people with schizophrenia or bipolar disorder.^51^ Shields et al reported that although most studies concluded that psychological interventions were cost-effective for people with schizophrenia or bipolar disorder, the incremental costs between psychological interventions and the comparators were highly uncertain. Jin et al and Shields et al reported that the reasons why different conclusions/results were reported by different studies might be methodological heterogeneity (e.g. use of different model structures, different sources of input data and different measures of health outcome) and conflict of interest (i.e. studies reported positive findings for an intervention manufactured by the sponsoring commercial companies).^49–51^

Specifically for this review, the reasons why different economic evaluations reported different conclusions for PAI might include: (a) patient heterogeneity – for example, Holt et al recruited only patients with schizophrenia and psychosis,^37^ whereas Verhaeghe et al^43^ and Meenan et al^39^ recruited patients with a mixed diagnosis of different mental health disorders; (b) use of different modalities of the intervention – for example, Holt et al assessed a group-based multicomponent lifestyle intervention used in the community setting,^37^ whereas Deenik et al assessed an individual-tailored multicomponent lifestyle intervention used in an in-patient setting;^42^ (c) widespread heterogeneity in the methods of economic evaluation – for example, Osborn et al assessed the 12-month cost-effectiveness of a multicomponent lifestyle intervention based on a clinical trial,^13^ whereas Verhaeghe et al assessed the 20-year cost-effectiveness of a multicomponent lifestyle intervention based on a clinical trial and an economic model;^43^ and (d) choice of different costing perspectives (i.e. which types of costs were included in the analysis) – according to a recently published systematic review of cost-of-illness studies,^52^ direct healthcare costs account for 11–87% of the total societal cost of schizophrenia; the rest 23–89% of the societal cost comprises productivity losses for patients and their carers, legal costs, sheltered housing and other costs. Of the nine studies included in this review, only three reported separate cost-effectiveness results from different costing perspectives.^37–39^ All three studies found that the adoption of a societal perspective made PAI less cost-effective compared with a narrow healthcare perspective^39^ or healthcare and social care perspective.^37,38^

The quality of the included studies ranges from moderate to high. The most common limitations which apply to most of the included studies are the use of a time horizon not long enough to reflect all important differences in costs and outcomes, and failure to test all important parameters whose values were uncertain in sensitivity analysis. Weight gain is associated with increased incidence of many chronic diseases, such as cardiovascular disease, diabetes and cancer. Therefore, any interventions that can effectively and continuously prevent or delay weight gain are likely to have a lifetime impact on patients’ future QALY gains and use of healthcare resources. Although it is usually not feasible to design a trial with a lifetime horizon, modelling methods can be used to extrapolate short-term trial outcomes over time to simulate the likely long-term impact of using an intervention based on the existing evidence. In the past 20 years, modelling methods have been routinely used by health technology assessment centres across the world (e.g. NICE and the Canadian Agency for Drugs and Technologies in Health (CADTH)) to explore the long-term cost-effectiveness of new interventions. Of the nine included studies, only two used a modelling method;^12,43^ and of these two studies, none modelled the lifetime outcomes of using a multicomponent lifestyle intervention including a PAI component. Therefore, the lifetime cost-effectiveness of such an intervention is still unknown and would require further research. Both trial- and model-based economic evaluations are subject to a number of potential selection biases and uncertainties. It is recommended that the impact of these biases and uncertainties should be assessed by comprehensive sensitivity analysis, such as deterministic sensitivity analysis and probabilistic/bootstrapping sensitivity analysis (which can be used to quantify the level of confidence in the results, in relation to the joint uncertainty of multiple parameters simultaneously).^15^ However, of the nine included studies, only one conducted adequate deterministic and probabilistic sensitivity analysis for both cost and effectiveness outcomes.^43^ Without the results of comprehensive sensitivity analyses, we cannot make judgements about the robustness of the study's conclusions.

It was noted that although the Drummond checklist emphasises the importance of adequately defining relevant patient subgroups, it does not address subgroups that were omitted. It is well documented that people with schizophrenia or bipolar disorder usually have their own unique combination of symptoms and may have very different responses to the same intervention.^8,9^ Therefore, an intervention that appeared to be cost-effective for one patient subgroup might not be cost-effective for another.^53^ It is recommended that potential patient subgroups should be considered early in the process of economic evaluation. Of the nine included studies, only two conducted subgroup analysis.^37,43^ It was acknowledged that subgroups might not always be relevant (e.g. if the trial inclusion/exclusion criteria are very strict and the recruited patient sample is fairly homogeneous) or feasible (e.g. the sample size is not powered to detect any difference in cost-effectiveness results across different subgroups). However, none of the included studies reported on their consideration of patient subgroups, such as potential subgroups considered, the rationale for conducting or not conducting subgroup analysis and any limitations.^53^

### Clinical relevance

The findings of our review suggest that PAIs should be used in combination with other lifestyle interventions (e.g. as a multicomponent lifestyle intervention), as currently there is a lack of economic evidence to support the use of PAIs alone. The mixed findings of the cost-effectiveness of multicomponent lifestyle interventions including a PAI component reported by included studies indicate that not all modalities of such an intervention are cost-effective for people with schizophrenia or bipolar disorder, and not all people with schizophrenia or bipolar disorder would benefit equally from the intervention. Although physical activity should be encouraged for all people with schizophrenia or bipolar disorder, owing to resource constraints of healthcare systems the provision of a multicomponent lifestyle intervention including a PAI component might need to be limited to those people who are mostly likely to benefit from it. Based on the limited evidence, it is suggested that the following patient subgroups might be more likely to benefit from a multicomponent lifestyle intervention including a PAI component: men;^43^ individuals with comorbid type 2 diabetes;^12^ and individuals who have been psychiatric hospital in-patients for ≥1 year.^42^ Further research is urgently needed to confirm the differential impacts of using multicomponent lifestyle interventions including a PAI component on different patient subgroups. When a multicomponent lifestyle intervention including a PAI component is provided to people with schizophrenia or bipolar disorder, it might need to be tailored to individual needs and used in combination with other approaches, such as adjustment of antipsychotic treatment, co-prescription with other drugs (e.g. metformin) and psychological interventions.

It should be noted that all included studies were conducted in high-income countries. As a result, the conclusions of the evidence might not be applicable to low- and middle-income countries (LMICs) owing to the differences in local economic situations and healthcare systems.

### Recommendations for future research

Based on the findings of this review, it is recommended that more economic evaluations of PAIs need to be conducted for people with schizophrenia or bipolar disorder in LMICs. It is also recommended that long-term follow-up studies are conducted to assess whether the effectiveness of PAIs can be maintained after 6–12 months.

Specific recommendations for future economic evaluations of PAIs for people with mental disorders are summarised as follows:

- compare the cost-effectiveness of different modalities of PAI, such as (i) group sessions versus individual sessions; (ii) PAIs with different intensity levels versus each other; and (iii) PAIs alone versus PAIs in combination with other interventions;
- explore the cost-effectiveness of PAIs for different patient subgroups, such as patients with different psychiatric diagnoses, different genders and different comorbidity status;
- use modelling methods to explore the lifetime cost-effectiveness of PAIs;
- report detailed information on costing data and calculation processes (e.g. detailed parameters and calculation tables) to facilitate assessment of quality, transparency, reproducibility and adaption of analyses to other settings/countries;
- consider potential patient subgroups early in the process of economic evaluation; any relevant patient subgroups considered but not included in the economic evaluation should be reported; and the rationale and limitations should be discussed.

### Strengths and limitations

#### Strengths

Although both NICE and the WHO recommend PAIs for people with schizophrenia or bipolar disorder, neither considered the cost-effectiveness evidence of these interventions. To our knowledge, our paper reports the first systematic review that summarised economic evidence on PAIs for people with schizophrenia or bipolar disorder. The findings of our review can be used to inform the recommendations of future clinical guidelines and commissioning decisions at national or local level. Based on the findings of our review, recommendations for future research are provided.

A comprehensive search strategy was developed to ensure that all relevant studies were captured. Searches were conducted on four mainstream databases and supplemented with forward and backward citations. Three reviewers were involved in the sifting process, and the inter-rater agreement score is reasonable.

#### Limitations

This review is subject to two main limitations. First, at the outset of the review, we intended to identify economic evidence on PAIs for all people with SMI. However, a pilot search on that topic returned over 9000 citations, which was not deemed to be manageable within the resource constraints of this project. Therefore, we decided to limit our review to people with more severe mental disorders – schizophrenia and bipolar disorder. This means that the findings of our review might not be directly applicable to people with other mental disorders. Second, this review only includes studies published in English, which may have limited the scope of the evidence identified. To assess the impact of this limitation, a language limit was not applied at the search stage. Of the abstracts retrieved from the search, only one study was excluded because it was reported in another language.^35^

## Data Availability

All data generated or analysed during this study are included in this published article.

## Supplementary material

For supplementary material accompanying this paper visit https://doi.org/10.1192/bjp.2023.52. click here to view supplementary material

## References

[1] Bhaskaran K, dos-Santos-Silva I, Leon DA, Douglas IJ, Smeeth L. Association of BMI with overall and cause-specific mortality: a population-based cohort study of 3⋅6 million adults in the UK. Lancet Diabetes Endocrinol 2018; 6: 944–53. 30389323 10.1016/S2213-8587(18)30288-2 PMC6249991

[2] de Hert M, Correll CU, Bobes J, Cetkovich-Bakmas M, Cohen D, Asai I, et al. Physical illness in patients with severe mental disorders. I. Prevalence, impact of medications and disparities in health care. World Psychiatry 2011; 10: 52–77. 21379357 10.1002/j.2051-5545.2011.tb00014.x PMC3048500

[3] Maina G, Salvi V, Vitalucci A, D'Ambrosio V, Bogetto F. Prevalence and correlates of overweight in drug-naïve patients with bipolar disorder. J Affect Disord 2007; 110: 149–55. 10.1016/j.jad.2007.12.233 18234351

[4] de Hert M, Detraux J, van Winkel R, Yu W, Correll CU. Metabolic and cardiovascular adverse effects associated with antipsychotic drugs. Nat Rev Endocrinol 2012; 8: 114–26. 10.1038/nrendo.2011.156 22009159

[5] Osborn DPJ, Nazareth I, King MB. Physical activity, dietary habits and coronary heart disease risk factor knowledge amongst people with severe mental illness: a cross sectional comparative study in primary care. Soc Psychiatry Psychiatr Epidemiol 2007; 42: 787–93. 17721669 10.1007/s00127-007-0247-3

[6] Afzal M, Siddiqi N, Ahmad B, Afsheen N, Aslam F, Ali A, et al. Prevalence of overweight and obesity in people with severe mental illness: systematic review and meta-analysis. Front Endocrinol (Lausanne) 2021; 12: 769309. 34899604 10.3389/fendo.2021.769309 PMC8656226

[7] Firth J, Siddiqi N, Koyanagi A, Siskind D, Rosenbaum S, Galletly C, et al. The Lancet Psychiatry Commission: a blueprint for protecting physical health in people with mental illness. Lancet Psychiatry 2019; 6: 675–712. 31324560 10.1016/S2215-0366(19)30132-4

[8] National Institute for Health and Care Excellence. Psychosis and Schizophrenia in Adults: Prevention and Management (Clinical Guideline CG178). NICE, 2014. 31869041

[9] National Institute for Health and Care Excellence. Bipolar Disorder: Assessment and Management (Clinical Guideline CG185). NICE, 2014. 31487127

[10] World Health Organization. Management of Physical Health Conditions in Adults with Severe Mental Disorders: WHO Guidelines. WHO, 2018. 30507109

[11] Verhaeghe N, de Maeseneer J, Maes L, van Heeringen C, Annemans L. Effectiveness and cost-effectiveness of lifestyle interventions on physical activity and eating habits in persons with severe mental disorders: a systematic review. Int J Behav Nutr Phys Act 2011; 8: 28. 21481247 10.1186/1479-5868-8-28 PMC3094265

[12] Park AL. Exploring the economic implications of a group-based lifestyle intervention for middle-aged adults with chronic schizophrenia and co-morbid type 2 diabetes. J Diabetes Metab 2014; 5(5): 1000366.

[13] Osborn D, Burton A, Hunter R, Marston L, Atkins L, Barnes T, et al. Clinical and cost-effectiveness of an intervention for reducing cholesterol and cardiovascular risk for people with severe mental illness in English primary care: a cluster randomised controlled trial. Lancet Psychiatry 2018; 5: 145–54. 10.1016/S2215-0366(18)30007-5 29396118

[14] Liberati A, Altman DG, Tetzlaff J, Mulrow C, Gøtzsche PC, Ioannidis JPA, et al. The PRISMA statement for reporting systematic reviews and meta-analyses of studies that evaluate healthcare interventions: explanation and elaboration. BMJ 2009; 339: b2700. 19622552 10.1136/bmj.b2700 PMC2714672

[15] Drummond M, Sculpher M, Claxton K, Stoddart G, Torrance G. Methods for the Economic Evaluation of Health Care Programmes (4th edn). Oxford University Press, 2015.

[16] Ouzzani M, Hammady H, Fedorowicz Z, Elmagarmid A. Rayyan – a web and mobile app for systematic reviews. Syst Rev 2016; 5(1): 210. 27919275 10.1186/s13643-016-0384-4 PMC5139140

[17] Park AL, McDaid D, Weiser P, Von Gottberg C, Becker T, Kilian R. Examining the cost effectiveness of interventions to promote the physical health of people with mental health problems: a systematic review. BMC Public Health 2013; 13: 787. 23988266 10.1186/1471-2458-13-787 PMC3765875

[18] Jin H, Li X. Combining cost-effectiveness results into a single measurement: What is the value? EClinicalMedicine 2022; 51:101563. 35860453 10.1016/j.eclinm.2022.101563 PMC9289627

[19] Popay J, Roberts H, Sowden A, Petticrew M, Arai L, Rodgers M, et al. Guidance on the Conduct of Narrative Synthesis in Systematic Reviews. A Product from the ESRC Methods Programme (Version 1). Lancaster University, 2006.

[20] Brozek JL, Canelo-Aybar C, Akl EA, Bowen JM, Bucher J, Chiu WA, et al. GRADE Guidelines 30: the GRADE approach to assessing the certainty of modeled evidence – An overview in the context of health decision-making. J Clin Epidemiol 2021; 129: 138–50. 32980429 10.1016/j.jclinepi.2020.09.018 PMC8514123

[21] Pezzot-Pearce TD, LeBow MD, Pearce JW. Increasing cost-effectiveness in obesity treatment through use of self-help behavioral manuals and decreased therapist contact. J Consult Clin Psychol. 1982; 50: 448–9. 6808042 10.1037//0022-006x.50.3.448

[22] Philipsson A, Duberg A, Möller M, Hagberg L. Cost-utility analysis of a dance intervention for adolescent girls with internalizing problems. Cost Eff Resour Alloc 2013; 11: 4. 23425608 10.1186/1478-7547-11-4 PMC3598394

[23] McRobbie H, Hajek P, Peerbux S, Kahan BC, Eldridge S, Trépel D, et al. Tackling obesity in areas of high social deprivation: clinical effectiveness and cost-effectiveness of a task-based weight management group programme – a randomised controlled trial and economic evaluation. Health Technol Assess 2016; 20(79): 1–150. 10.3310/hta20790 PMC5107886 27802843

[24] Wilson DK, Lorig K, Klein WMP, Riley W, Sweeney AM, Christensen A. Efficacy and cost-effectiveness of behavioral interventions in nonclinical settings for improving health outcomes. Health Psychol 2019; 38: 689–700. 31368753 10.1037/hea0000773

[25] Richards DA, Bower P, Chew-Graham C, Gask L, Lovell K, Cape J, et al. Clinical effectiveness and cost-effectiveness of collaborative care for depression in UK primary care (CADET): a cluster randomised controlled trial. Health Technol Assess 2016; 20(14): 1–192. 10.3310/hta20140 PMC4809468 26910256

[26] Galbraith N, Rose C, Rose P. The roles of motivational interviewing and self-efficacy on outcomes and cost-effectiveness of a community-based exercise intervention for inactive middle-older aged adults. Health Soc Care Community 2022; 30(4): e1048–60. 34260782 10.1111/hsc.13510

[27] Böge K, Hahne I, Bergmann N, Wingenfeld K, Zierhut M, Thomas N, et al. Mindfulness-based group therapy for in-patients with schizophrenia spectrum disorders – Feasibility, acceptability, and preliminary outcomes of a rater-blinded randomized controlled trial. Schizophr Res 2021; 228: 134–44. 33434727 10.1016/j.schres.2020.12.008

[28] Ayenew W, Gathright EC, Coffey EM, Courtney A, Rogness J, Busch AM. Feasibility of a bike-share program in adults with serious mental illness enrolled in an outpatient psychiatric rehabilitation program. J Phys Act Health 2019; 16: 380–3. 30925847 10.1123/jpah.2018-0233 PMC6916713

[29] Richardson CR, Avripas SA, Neal DL, Marcus SM. Increasing lifestyle physical activity in patients with depression or other serious mental illness. J Psychiatr Pract 2005; 11: 379–88. 16304506 10.1097/00131746-200511000-00004

[30] Kandola AA, Osborn DPJ. Physical activity as an intervention in severe mental illness. BJPsych Adv 2022; 28: 112–21.

[31] Stumbo SP, Yarborough BJH, Yarborough MT, Janoff SL, Stevens VJ, Lewinsohn M, et al. Costs of implementing a behavioral weight-loss and lifestyle-change program for individuals with serious mental illnesses in community settings. Transl Behav Med 2015; 5: 269–76. 26327932 10.1007/s13142-015-0322-3 PMC4537454

[32] Walburg FS, de Joode JW, Brandt HE, van Tulder MW, Aiaanse MC, van Meijel B. Implementation of a lifestyle intervention for people with a severe mental illness (SMILE): a process evaluation. BMC Health Serv Res 2022; 22(1): 27. 34983508 10.1186/s12913-021-07391-3 PMC8729040

[33] Maurus I, Hasan A, Schmitt A, Roeh A, Keeser D, Malchow B, et al. Aerobic endurance training to improve cognition and enhance recovery in schizophrenia: design and methodology of a multicenter randomized controlled trial. Eur Arch Psychiatry Clin Neurosci 2020; 271: 315–24. 32748261 10.1007/s00406-020-01175-2 PMC8257533

[34] Lewis M, Chondros P, Mihalopoulos C, Lee YY, Gunn JM, Harvey C, et al. The assertive cardiac care trial: a randomised controlled trial of a coproduced assertive cardiac care intervention to reduce absolute cardiovascular disease risk in people with severe mental illness in the primary care setting. Contemp Clin Trials 2020; 97:106143. 32931919 10.1016/j.cct.2020.106143

[35] Duberg A, Möller M, Taube J. Dans kan ge unga skydd mot psykisk ohälsa [Dance can serve as a protective factor in preventing mental illness]. Läkartidningen 2013; 110: CDTT. 24163907

[36] Landis JR, Koch GG. The measurement of observer agreement for categorical data. Biometrics. 1977; 33: 159–74. 843571

[37] Holt RIG, Gossage-Worrall R, Hind D, Bradburn MJ, McCrone P, Morris T, et al. Structured lifestyle education for people with schizophrenia, schizoaffective disorder and first-episode psychosis (STEPWISE): randomised controlled trial. Br J Psychiatry 2019; 214: 63–73. 30251622 10.1192/bjp.2018.167 PMC6330076

[38] Heslin M, Patel A, Stahl D, Gardner-Sood P, Mushore M, Smith S, et al. Randomised controlled trial to improve health and reduce substance use in established psychosis (IMPaCT): cost-effectiveness of integrated psychosocial health promotion. BMC Psychiatry 2017; 17(1): 407. 29273021 10.1186/s12888-017-1570-1 PMC5741948

[39] Meenan RT, Stumbo SP, Yarborough MT, Leo MC, Yarborough BJH, Green CA. An economic evaluation of a weight loss intervention program for people with serious mental illnesses taking antipsychotic medications. Admin Policy Ment Health 2016; 43: 604–15. 10.1007/s10488-015-0669-2 PMC4705000 26149243

[40] Janssen EM, Jerome GJ, Dalcin AT, Gennusa Jv., Goldsholl S, Frick KD, et al. A cost analysis of implementing a behavioral weight loss intervention in community mental health settings: results from the ACHIEVE trial. Obesity 2017; 25: 1006–13. 28398006 10.1002/oby.21836 PMC5445002

[41] Looijmans A, Jörg F, Bruggeman R, Schoevers RA, Corpeleijn E, Feenstra TL, et al. Cost-effectiveness and budget impact of a lifestyle intervention to improve cardiometabolic health in patients with severe mental illness. Glob Reg Health Technol Assess 2020; 7: 131–8. 36627968 10.33393/grhta.2020.2027 PMC9677596

[42] Deenik J, van Lieshout C, van Driel HF, Frederix GWJ, Hendriksen IJM, van Harten PN, et al. Cost-effectiveness of a multidisciplinary lifestyle-enhancing treatment for inpatients with severe mental illness: the MULTI study. Schizophr Bull Open 2022; 3(1): sgac022. 10.1093/schizbullopen/sgac022 PMC11206082 39144774

[43] Verhaeghe N, de Smedt D, de Maeseneer J, Maes L, van Heeringen C, Annemans L. Cost-effectiveness of health promotion targeting physical activity and healthy eating in mental health care. BMC Public Health 2014; 14: 856. 25134636 10.1186/1471-2458-14-856 PMC4150981

[44] Daumit GL, Dickerson FB, Wang NY, Dalcin A, Jerome GJ, Anderson CAM, et al. A behavioral weight-loss intervention in persons with serious mental illness. New Eng J Med 2013; 368: 1594–602. 23517118 10.1056/NEJMoa1214530 PMC3743095

[45] Yarborough BJH, Leo MC, Stumbo S, Perrin NA, Green CA. STRIDE: a randomized trial of a lifestyle intervention to promote weight loss among individuals taking antipsychotic medications. BMC Psychiatry 2013; 13: 238. 24074269 10.1186/1471-244X-13-238 PMC3907020

[46] Verhaeghe N, Clays E, Vereecken C, de Maeseneer J, Maes L, van Heeringen C, et al. Health promotion in individuals with mental disorders: A cluster preference randomized controlled trial. BMC Public Health 2013; 13: 657. 23855449 10.1186/1471-2458-13-657 PMC3721998

[47] Looijmans A, Jörg F, Bruggeman R, Schoevers RA, Corpeleijn E. Multimodal lifestyle intervention using a web-based tool to improve cardiometabolic health in patients with serious mental illness: results of a cluster randomized controlled trial (LION). BMC Psychiatry 2019; 19: 339. 31690281 10.1186/s12888-019-2310-5 PMC6833253

[48] Deenik J, Tenback DE, Tak ECPM, Rutters F, Hendriksen IJM, van Harten PN. Changes in physical and psychiatric health after a multidisciplinary lifestyle enhancing treatment for inpatients with severe mental illness: the MULTI study I. Schizophr Res 2019; 204: 360–7. 30055884 10.1016/j.schres.2018.07.033

[49] Jin H, Tappenden P, Robinson S, Achilla E, MacCabe JH, Aceituno D, et al. A systematic review of economic models across the entire schizophrenia pathway. PharmacoEconomics 2020; 38: 537–55. 32144726 10.1007/s40273-020-00895-6

[50] Jin H, Tappenden P, Robinson S, Achilla E, Aceituno D, Byford S. Systematic review of the methods of health economic models assessing antipsychotic medication for schizophrenia. PLoS One 2020; 15(7): e0234996. 32649663 10.1371/journal.pone.0234996 PMC7351140

[51] Shields GE, Buck D, Elvidge J, Hayhurst KP, Davies LM. Cost-effectiveness evaluations of psychological therapies for schizophrenia and bipolar disorder: a systematic review. Int J Technol Assess Health Care 2019; 35: 317–26. 31328702 10.1017/S0266462319000448 PMC6707812

[52] Lin C, Zhang X, Jin H. The societal cost of schizophrenia: an updated systematic review of cost-of-illness studies. Pharmacoeconomics [Internet] 2023; 41:139–53. 36404364 10.1007/s40273-022-01217-8

[53] Shields GE, Wilberforce M, Clarkson P, Farragher T, Verma A, Davies LM. Factors limiting subgroup analysis in cost-effectiveness analysis and a call for transparency. Pharmacoeconomics 2022; 40: 149–56. 34713422 10.1007/s40273-021-01108-4 PMC8553493

