# Supplementary material for: Cost-effectiveness analysis of physical activity interventions for people with schizophrenia or bipolar disorder: systematic review
Source: Br J Psychiatry. 2023 Aug;223(2):362–76. doi: 10.1192/bjp.2023.52 (PMC10405045; doi:10.1192/bjp.2023.52)
Supplement: Supplementary file 1 [file S0007125023000521sup001.docx]

**Supplementary material**

This supplementary material contains five sections:

- Section 1 reports the PRISMA table
- Section 2 reports the search strategy and results
- Section 3 lists all included studies
- Section 4 summarise the results of quality assessment for included studies
- Section 5 presents the GRADE table for included studies

The protocol for the systematic review was registered on the National Institute of Health Research Database and can be found online (www.crd.york.ac.uk/prospero, CRD42022359492).

**Section 1: PRISMA table**

**Table 1. PRISMA 2020 checklist**

| **Section and Topic** | **Item #** | **Checklist item** | **Location where item is reported** |
| --- | --- | --- | --- |
| **TITLE** | | |  |
| Title | 1 | Identify the report as a systematic review. |  |
| **ABSTRACT** | | |  |
| Abstract | 2 | See the PRISMA 2020 for Abstracts checklist. |  |
| **INTRODUCTION** | | |  |
| Rationale | 3 | Describe the rationale for the review in the context of existing knowledge. |  |
| Objectives | 4 | Provide an explicit statement of the objective(s) or question(s) the review addresses. | Manuscript p3-4, line 48-65 |
| **METHODS** | | |  |
| Eligibility criteria | 5 | Specify the inclusion and exclusion criteria for the review and how studies were grouped for the syntheses. |  |
| Information sources | 6 | Specify all databases, registers, websites, organisations, reference lists and other sources searched or consulted to identify studies. Specify the date when each source was last searched or consulted. | Manuscript p3-4, line 82-93 |
| Search strategy | 7 | Present the full search strategies for all databases, registers and websites, including any filters and limits used. | Manuscript p4, line 97-102. |
| Selection process | 8 | Specify the methods used to decide whether a study met the inclusion criteria of the review, including how many reviewers screened each record and each report retrieved, whether they worked independently, and if applicable, details of automation tools used in the process. | Supplementary material Section 2. |
| Data collection process | 9 | Specify the methods used to collect data from reports, including how many reviewers collected data from each report, whether they worked independently, any processes for obtaining or confirming data from study investigators, and if applicable, details of automation tools used in the process. | Manuscript p4 line 105-112 |
| Data items | 10a | List and define all outcomes for which data were sought. Specify whether all results that were compatible with each outcome domain in each study were sought (e.g. for all measures, time points, analyses), and if not, the methods used to decide which results to collect. | Manuscript p4 line 115-116 |
|  | 10b | List and define all other variables for which data were sought (e.g. participant and intervention characteristics, funding sources). Describe any assumptions made about any missing or unclear information. | Manuscript p4-5 line 116-123 |
| Study risk of bias assessment | 11 | Specify the methods used to assess risk of bias in the included studies, including details of the tool(s) used, how many reviewers assessed each study and whether they worked independently, and if applicable, details of automation tools used in the process. | Manuscript p4-5 line 116-123 |
| Effect measures | 12 | Specify for each outcome the effect measure(s) (e.g. risk ratio, mean difference) used in the synthesis or presentation of results. | Manuscript p5 line 125-135 |
| Synthesis methods | 13a | Describe the processes used to decide which studies were eligible for each synthesis (e.g. tabulating the study intervention characteristics and comparing against the planned groups for each synthesis (item #5)). | Manuscript p4-5 line 116-123 |
|  | 13b | Describe any methods required to prepare the data for presentation or synthesis, such as handling of missing summary statistics, or data conversions. | Manuscript p5, line 137-148 |
|  | 13c | Describe any methods used to tabulate or visually display results of individual studies and syntheses. | Manuscript p5, line 137-148 |
|  | 13d | Describe any methods used to synthesize results and provide a rationale for the choice(s). If meta-analysis was performed, describe the model(s), method(s) to identify the presence and extent of statistical heterogeneity, and software package(s) used. | Manuscript p5, line 137-148 |
|  | 13e | Describe any methods used to explore possible causes of heterogeneity among study results (e.g. subgroup analysis, meta-regression). | Manuscript p5, line 137-148 |
|  | 13f | Describe any sensitivity analyses conducted to assess robustness of the synthesized results. | N/A |
| Reporting bias assessment | 14 | Describe any methods used to assess risk of bias due to missing results in a synthesis (arising from reporting biases). | N/A |
| Certainty assessment | 15 | Describe any methods used to assess certainty (or confidence) in the body of evidence for an outcome. | Manuscript p5, line 137-148 |
| **RESULTS** | | |  |
| Study selection | 16a | Describe the results of the search and selection process, from the number of records identified in the search to the number of studies included in the review, ideally using a flow diagram. |  |
|  | 16b | Cite studies that might appear to meet the inclusion criteria, but which were excluded, and explain why they were excluded. | Manuscript p6 line 151-164; Figure 1. |
| Study characteristics | 17 | Cite each included study and present its characteristics. | N/A |
| Risk of bias in studies | 18 | Present assessments of risk of bias for each included study. | Manuscript Table 1. |
| Results of individual studies | 19 | For all outcomes, present, for each study: (a) summary statistics for each group (where appropriate) and (b) an effect estimate and its precision (e.g. confidence/credible interval), ideally using structured tables or plots. | Fig 2. |
| Results of syntheses | 20a | For each synthesis, briefly summarise the characteristics and risk of bias among contributing studies. | Table 2. |
|  | 20b | Present results of all statistical syntheses conducted. If meta-analysis was done, present for each the summary estimate and its precision (e.g. confidence/credible interval) and measures of statistical heterogeneity. If comparing groups, describe the direction of the effect. | Supplementary material Section 4 |
|  | 20c | Present results of all investigations of possible causes of heterogeneity among study results. | N/A |
|  | 20d | Present results of all sensitivity analyses conducted to assess the robustness of the synthesized results. | Supplementary material A Section 4 |
| Reporting biases | 21 | Present assessments of risk of bias due to missing results (arising from reporting biases) for each synthesis assessed. | N/A |
| Certainty of evidence | 22 | Present assessments of certainty (or confidence) in the body of evidence for each outcome assessed. | Supplementary material A Section 4 |
| **DISCUSSION** | | |  |
| Discussion | 23a | Provide a general interpretation of the results in the context of other evidence. |  |
|  | 23b | Discuss any limitations of the evidence included in the review. | Manuscript p13-14, line 377-390 |
|  | 23c | Discuss any limitations of the review processes used. | Manuscript p14-15, line 409-444 |
|  | 23d | Discuss implications of the results for practice, policy, and future research. | Manuscript p17-18, line 499-508. |
| **OTHER INFORMATION** | | |  |
| Registration and protocol | 24a | Provide registration information for the review, including register name and registration number, or state that the review was not registered. |  |
|  | 24b | Indicate where the review protocol can be accessed, or state that a protocol was not prepared. | Manuscript p3, line 79-80 |
|  | 24c | Describe and explain any amendments to information provided at registration or in the protocol. | Manuscript p3, line 79-80 |
| Support | 25 | Describe sources of financial or non-financial support for the review, and the role of the funders or sponsors in the review. | N/A |
| Competing interests | 26 | Declare any competing interests of review authors. | Manuscript p18, line 517-518 |
| Availability of data, code and other materials | 27 | Report which of the following are publicly available and where they can be found: template data collection forms; data extracted from included studies; data used for all analyses; analytic code; any other materials used in the review. | Manuscript p18, line 520-521 |

**Section 2: Electronic search strategies**

**Table 2. Medline search terms and results**

|  | **MEDLINE SEARCH TERMS:** Ovid MEDLINE(R) ALL <1946 to July 05, 2022> | **Number of hits** (Results from 6 Jul 2022) |
| --- | --- | --- |
| 1 | exp "schizophrenia spectrum and other psychotic disorders"/ | 159,003 |
| 2 | exp psychotic disorders/ or exp perceptual disorders/ or delusions/ or hallucinations/ or speech disorders/ or catatonia/ or paranoid disorders/ | 105,089 |
| 3 | (schizo$ or psychotic$ or psychosis or psychoses or ((thinking or thought) adj2 (disorder$ or disturbance$ or problem$)) or delusion$ or catatoni$ or hallucinat$ or hebephreni$ or oligophreni$ or paranoi$).mp. | 268,263 |
| 4 | ((chronic$ or long term or persistent or serious$ or sever$) adj2 (mental$ or psychiatric or psycho$) adj2 (ill$ or disorder$ or disease$ or problem$ or disturb$ or disable$)).mp. | 22,205 |
| 5 | exp bipolar disorder/ | 43,904 |
| 6 | ((bipolar or bi polar) adj5 (disorder$ or depress$)).tw. | 39,138 |
| 7 | (((cyclothymi$ or rapid or ultradian) adj5 cycl$) or RCBD).tw. | 7,234 |
| 8 | (hypomania$ or mania$ or manic$ or schizoaffective).tw. | 26,893 |
| 9 | 1 or 2 or 3 or 4 or 5 or 6 or 7 or 8 | 355,514 |
| 10 | exp Cost-Benefit Analysis/ | 90,099 |
| 11 | *economic evaluation/ or exp "cost benefit analysis"/ or exp "cost effectiveness analysis"/ or exp "cost minimization analysis"/ or exp "cost utility analysis"/ | 258,875 |
| 12 | exp cost-effectiveness/ | 90,099 |
| 13 | (cost$ adj2 (effect$ or benefit$ or utility or utilities or outcome$ or consequence$)).mp. [mp=title, abstract, original title, name of substance word, subject heading word, floating sub-heading word, keyword heading word, organism supplementary concept word, protocol supplementary concept word, rare disease supplementary concept word, unique identifier, synonyms] | 231,659 |
| 14 | (cost$ adj minimi$).mp. [mp=title, abstract, original title, name of substance word, subject heading word, floating sub-heading word, keyword heading word, organism supplementary concept word, protocol supplementary concept word, rare disease supplementary concept word, unique identifier, synonyms] | 1,655 |
| 15 | cost* analys?s.mp. [mp=title, abstract, original title, name of substance word, subject heading word, floating sub-heading word, keyword heading word, organism supplementary concept word, protocol supplementary concept word, rare disease supplementary concept word, unique identifier, synonyms] | 58,395 |
| 16 | 10 or 11 or 12 or 13 or 14 or 15 | 392,549 |
| 17 | exp Weight Reduction Programs/ | 2,959 |
| 18 | weight reduc* program*.tw. | 729 |
| 19 | exp Weight Loss/ | 47,533 |
| 20 | weight loss.tw. | 99,559 |
| 21 | exp body weight changes/ | 80,481 |
| 22 | exp Exercise/ | 233,384 |
| 23 | exercise.tw. | 291,802 |
| 24 | exp Exercise Movement Techniques/ | 9,781 |
| 25 | exp Exercise Therapy/ | 60,113 |
| 26 | exercise therap*.tw. | 4,082 |
| 27 | exp "Physical Education and Training"/ | 14,019 |
| 28 | ((physical adj6 education) and training).tw. | 1,467 |
| 29 | (physical and (education adj6 training)).tw. | 1,904 |
| 30 | ((physical adj6 education) and training).tw. | 1,467 |
| 31 | (physical and (education adj6 training)).tw. | 1,904 |
| 32 | exp Physical Fitness/ | 35,068 |
| 33 | physical fitness.tw. | 11,066 |
| 34 | (physical adj6 fitness).tw. | 14,302 |
| 35 | Physical Exertion/ | 57,310 |
| 36 | exertion.tw. | 18,831 |
| 37 | exp Sports/ | 207,345 |
| 38 | sport*.tw. | 90,282 |
| 39 | Motor Activity/ | 99,729 |
| 40 | physical activit*.tw. | 135,053 |
| 41 | (physical adj6 activit*).tw. | 145,020 |
| 42 | exp Walking/ | 63,892 |
| 43 | walk*.tw. | 136,163 |
| 44 | exp Jogging/ | 837 |
| 45 | jog*.tw. | 2,629 |
| 46 | exp Swimming/ | 26,448 |
| 47 | swim*.tw. | 45,095 |
| 48 | Bicycling/ | 12,460 |
| 49 | bicycl*.tw. | 26,401 |
| 50 | weight training.tw. | 1,043 |
| 51 | (weight adj6 training).tw. | 3,982 |
| 52 | Dancing/ | 3,338 |
| 53 | Dance Therapy/ | 430 |
| 54 | danc*.tw. | 8,601 |
| 55 | (dance adj6 therap*).tw. | 336 |
| 56 | dance therap*.tw. | 127 |
| 57 | (aerobic adj6 exercis*).tw. | 15,441 |
| 58 | aerobic exercise.tw. | 10,974 |
| 59 | Resistance Training/ | 11,369 |
| 60 | resistance train*.tw. | 10,142 |
| 61 | ((resistance or strength) and train*).tw. | 42,329 |
| 62 | ((resistance or strength) adj6 train*).tw. | 20,858 |
| 63 | strength train*.tw. | 5,829 |
| 64 | body weight management.tw. | 207 |
| 65 | (weight adj6 manag*).tw. | 10,966 |
| 66 | weight loss intervention.tw. | 1,439 |
| 67 | ((weight and loss) adj6 intervention*).tw. | 8,708 |
| 68 | ((weight adj6 loss) and intervention*).tw. | 16,739 |
| 69 | exp Behavior Therapy/ | 85,750 |
| 70 | behaviour therap*.tw. | 2,644 |
| 71 | (behaviour adj6 therap*).tw. | 4,108 |
| 72 | exp Cognitive Therapy/ | 34,440 |
| 73 | cogniti* therap*.tw. | 3,471 |
| 74 | (cogniti* adj6 therap*).tw. | 31,984 |
| 75 | exp Psychotherapy/ | 212,728 |
| 76 | lifestyle modification.tw. | 4,923 |
| 77 | (lifestyle adj6 modif*).tw. | 11,175 |
| 78 | 17 or 18 or 19 or 20 or 21 or 22 or 23 or 24 or 25 or 26 or 27 or 28 or 29 or 30 or 31 or 32 or 33 or 34 or 35 or 36 or 37 or 38 or 39 or 40 or 41 or 42 or 43 or 44 or 45 or 46 or 47 or 48 or 49 or 50 or 51 or 52 or 53 or 54 or 55 or 56 or 57 or 58 or 59 or 60 or 61 or 62 or 63 or 64 or 65 or 66 or 67 or 68 or 69 or 70 or 71 or 72 or 73 or 74 or 75 or 76 or 77 | 1,253,664 |
| 79 | 9 and 16 and 78 | 637 |
| 80 | remove duplicates from 79 | **633** |

**Table 3. EMBASE search terms and results**

|  | **EMBASE SEARCH TERMS: <1974 to 2022 July 05>** | **Number of hits** (Results from 6 Jul 2022) |
| --- | --- | --- |
| 1 | exp schizophrenia/ | 193,288 |
| 2 | exp psychotic disorders/ or exp perceptual disorders/ or delusions/ or hallucinations/ or speech disorders/ or catatonia/ or paranoid disorders/ | 351,295 |
| 3 | (schizo$ or psychotic$ or psychosis or psychoses or ((thinking or thought) adj2 (disorder$ or disturbance$ or problem$)) or delusion$ or catatoni$ or hallucinat$ or hebephreni$ or oligophreni$ or paranoi$).mp. | 381,714 |
| 4 | ((chronic$ or long term or persistent or serious$ or sever$) adj2 (mental$ or psychiatric or psycho$) adj2 (ill$ or disorder$ or disease$ or problem$ or disturb$ or disable$)).mp. | 29,239 |
| 5 | exp bipolar disorder/ or *schizoaffective psychosis/ | 73,874 |
| 6 | ((bipolar or bi polar) adj5 (disorder$ or depress$)).tw. | 59,343 |
| 7 | (((cyclothymi$ or rapid or ultradian) adj5 cycl$) or RCBD).tw. | 9,330 |
| 8 | (hypomania$ or mania$ or manic$ or schizoaffective).tw. | 37,333 |
| 9 | 1 or 2 or 3 or 4 or 5 or 6 or 7 or 8 | 502,071 |
| 10 | exp Cost-Benefit Analysis/ | 90,813 |
| 11 | *economic evaluation/ or exp "cost benefit analysis"/ or exp "cost effectiveness analysis"/ or exp "cost minimization analysis"/ or exp "cost utility analysis"/ | 257,993 |
| 12 | exp cost-effectiveness/ | 169,001 |
| 13 | (cost$ adj2 (effect$ or benefit$ or utility or utilities or outcome$ or consequence$)).mp. [mp=title, abstract, heading word, drug trade name, original title, device manufacturer, drug manufacturer, device trade name, keyword heading word, floating subheading word, candidate term word] | 390,225 |
| 14 | (cost$ adj minimi$).mp. [mp=title, abstract, heading word, drug trade name, original title, device manufacturer, drug manufacturer, device trade name, keyword heading word, floating subheading word, candidate term word] | 4,832 |
| 15 | cost* analys?s.mp. [mp=title, abstract, heading word, drug trade name, original title, device manufacturer, drug manufacturer, device trade name, keyword heading word, floating subheading word, candidate term word] | 18,171 |
| 16 | 10 or 11 or 12 or 13 or 14 or 15 | 398,908 |
| 17 | exp Weight Reduction Programs/ | 2,919 |
| 18 | weight reduc* program*.tw. | 981 |
| 19 | exp Weight Loss/ | 67,136 |
| 20 | weight loss.tw. | 161,779 |
| 21 | exp body weight changes/ | 101,303 |
| 22 | exp Exercise/ | 395,093 |
| 23 | exercise.tw. | 390,899 |
| 24 | exp Exercise Movement Techniques/ | 91,443 |
| 25 | exp Exercise Therapy/ | 91,443 |
| 26 | exercise therap*.tw. | 5,619 |
| 27 | exp "Physical Education and Training"/ | 12,587 |
| 28 | ((physical adj6 education) and training).tw. | 2,223 |
| 29 | (physical and (education adj6 training)).tw. | 2,795 |
| 30 | ((physical adj6 education) and training).tw. | 2,223 |
| 31 | (physical and (education adj6 training)).tw. | 2,795 |
| 32 | exp Physical Fitness/ | 40,054 |
| 33 | physical fitness.tw. | 13,682 |
| 34 | (physical adj6 fitness).tw. | 17,708 |
| 35 | Physical Exertion/ | 284,367 |
| 36 | exertion.tw. | 25,823 |
| 37 | exp Sports/ | 195,321 |
| 38 | sport*.tw. | 118,183 |
| 39 | Motor Activity/ | 46,403 |
| 40 | physical activit*.tw. | 182,151 |
| 41 | (physical adj6 activit*).tw. | 196,204 |
| 42 | exp Walking/ | 138,358 |
| 43 | walk*.tw. | 190,843 |
| 44 | exp Jogging/ | 2,347 |
| 45 | jog*.tw. | 3,237 |
| 46 | exp Swimming/ | 25,338 |
| 47 | swim*.tw. | 53,368 |
| 48 | Bicycling/ | 13,247 |
| 49 | bicycl*.tw. | 34,585 |
| 50 | weight training.tw. | 1,226 |
| 51 | (weight adj6 training).tw. | 5,185 |
| 52 | Dancing/ | 5,850 |
| 53 | Dance Therapy/ | 647 |
| 54 | danc*.tw. | 11,107 |
| 55 | (dance adj6 therap*).tw. | 705 |
| 56 | dance therap*.tw. | 252 |
| 57 | (aerobic adj6 exercis*).tw. | 21,704 |
| 58 | aerobic exercise.tw. | 15,404 |
| 59 | Resistance Training/ | 23,806 |
| 60 | resistance train*.tw. | 12,022 |
| 61 | ((resistance or strength) and train*).tw. | 55,926 |
| 62 | ((resistance or strength) adj6 train*).tw. | 26,143 |
| 63 | strength train*.tw. | 7,539 |
| 64 | body weight management.tw. | 267 |
| 65 | (weight adj6 manag*).tw. | 16,017 |
| 66 | weight loss intervention.tw. | 2,007 |
| 67 | ((weight and loss) adj6 intervention*).tw. | 13,066 |
| 68 | ((weight adj6 loss) and intervention*).tw. | 26,536 |
| 69 | exp Behavior Therapy/ | 66,873 |
| 70 | behaviour therap*.tw. | 3,831 |
| 71 | (behaviour adj6 therap*).tw. | 6,032 |
| 72 | exp Cognitive Therapy/ | 66,694 |
| 73 | cogniti* therap*.tw. | 5,483 |
| 74 | (cogniti* adj6 therap*).tw. | 45,491 |
| 75 | exp Psychotherapy/ | 275,575 |
| 76 | lifestyle modification.tw. | 7,465 |
| 77 | (lifestyle adj6 modif*).tw. | 16,559 |
| 78 | 17 or 18 or 19 or 20 or 21 or 22 or 23 or 24 or 25 or 26 or 27 or 28 or 29 or 30 or 31 or 32 or 33 or 34 or 35 or 36 or 37 or 38 or 39 or 40 or 41 or 42 or 43 or 44 or 45 or 46 or 47 or 48 or 49 or 50 or 51 or 52 or 53 or 54 or 55 or 56 or 57 or 58 or 59 or 60 or 61 or 62 or 63 or 64 or 65 or 66 or 67 or 68 or 69 or 70 or 71 or 72 or 73 or 74 or 75 or 76 or 77 | 1,652,436 |
| 79 | 9 and 16 and 78 | 978 |
| 80 | remove duplicates from 79 | **967** |

**Table 4. PsycINFO search terms and results**

|  | **PsycINFO SEARCH TERMS <1806 to June Week 4 2022>** | **Number of hits** (Results from 6 Jul 2022) |
| --- | --- | --- |
| 1 | exp schizophrenia/ | 96,148 |
| 2 | exp psychosis/ or exp thought disturbances/ or exp delusions/ or exp hallucinations/ or exp speech disorders/ or exp catatonia/ or exp paranoia/ | 177,407 |
| 3 | (schizo$ or psychotic$ or psychosis or psychoses or ((thinking or thought) adj2 (disorder$ or disturbance$ or problem$)) or delusion$ or catatoni$ or hallucinat$ or hebephreni$ or oligophreni$ or paranoi$).mp. | 230,043 |
| 4 | ((chronic$ or long term or persistent or serious$ or sever$) adj2 (mental$ or psychiatric or psycho$) adj2 (ill$ or disorder$ or disease$ or problem$ or disturb$ or disable$)).mp. | 25,364 |
| 5 | exp bipolar disorder/ or *Schizoaffective Disorder/ | 34,964 |
| 6 | ((bipolar or bi polar) adj5 (disorder$ or depress$)).tw. | 37,635 |
| 7 | (((cyclothymi$ or rapid or ultradian) adj5 cycl$) or RCBD).tw. | 2,970 |
| 8 | (hypomania$ or mania$ or manic$ or Schizoaffective).tw. | 29,329 |
| 9 | 1 or 2 or 3 or 4 or 5 or 6 or 7 or 8 | 319,171 |
| 10 | exp cost-effectiveness/ | 46,906 |
| 11 | (cost$ adj2 (effect$ or benefit$ or utility or utilities or outcome$ or consequence$)).mp. [mp=title, abstract, heading word, table of contents, key concepts, original title, tests & measures, mesh word] | 30,096 |
| 12 | (cost$ adj minimi$).mp. [mp=title, abstract, heading word, table of contents, key concepts, original title, tests & measures, mesh word] | 179 |
| 13 | cost* analys?s.mp. [mp=title, abstract, heading word, table of contents, key concepts, original title, tests & measures, mesh word] | 20,203 |
| 14 | 10 or 11 or 12 or 13 | 68,971 |
| 15 | weight reduc* program*.tw. | 282 |
| 16 | exp Weight Loss/ | 4,326 |
| 17 | weight loss.tw. | 12,690 |
| 18 | exp Exercise/ | 30,055 |
| 19 | exercise.tw. | 57,981 |
| 20 | exercise therap*.tw. | 573 |
| 21 | ((physical adj6 education) and training).tw. | 1,112 |
| 22 | (physical and (education adj6 training)).tw. | 1,221 |
| 23 | ((physical adj6 education) and training).tw. | 1,112 |
| 24 | (physical and (education adj6 training)).tw. | 1,221 |
| 25 | exp Physical Fitness/ | 4,699 |
| 26 | physical fitness.tw. | 3,480 |
| 27 | (physical adj6 fitness).tw. | 4,698 |
| 28 | exertion.tw. | 3,130 |
| 29 | exp Sports/ | 38,376 |
| 30 | sport*.tw. | 40,603 |
| 31 | physical activit*.tw. | 41,652 |
| 32 | (physical adj6 activit*).tw. | 45,526 |
| 33 | exp Walking/ | 6,492 |
| 34 | walk*.tw. | 30,795 |
| 35 | jog*.tw. | 766 |
| 36 | exp Swimming/ | 2,024 |
| 37 | swim*.tw. | 10,766 |
| 38 | bicycl*.tw. | 2,587 |
| 39 | weight training.tw. | 232 |
| 40 | (weight adj6 training).tw. | 828 |
| 41 | Dance Therapy/ | 1,326 |
| 42 | danc*.tw. | 9,416 |
| 43 | (dance adj6 therap*).tw. | 1,511 |
| 44 | dance therap*.tw. | 596 |
| 45 | (aerobic adj6 exercis*).tw. | 2,966 |
| 46 | aerobic exercise.tw. | 2,408 |
| 47 | resistance train*.tw. | 888 |
| 48 | ((resistance or strength) and train*).tw. | 10,186 |
| 49 | ((resistance or strength) adj6 train*).tw. | 2,840 |
| 50 | strength train*.tw. | 740 |
| 51 | body weight management.tw. | 23 |
| 52 | (weight adj6 manag*).tw. | 3,335 |
| 53 | weight loss intervention.tw. | 463 |
| 54 | ((weight and loss) adj6 intervention*).tw. | 2,082 |
| 55 | ((weight adj6 loss) and intervention*).tw. | 3,724 |
| 56 | exp Behavior Therapy/ | 22,575 |
| 57 | behaviour therap*.tw. | 3,360 |
| 58 | (behaviour adj6 therap*).tw. | 4,172 |
| 59 | exp Cognitive Therapy/ | 13,846 |
| 60 | cogniti* therap*.tw. | 8,254 |
| 61 | (cogniti* adj6 therap*).tw. | 44,886 |
| 62 | exp Psychotherapy/ | 215,371 |
| 63 | lifestyle modification.tw. | 594 |
| 64 | (lifestyle adj6 modif*).tw. | 1,449 |
| 65 | 15 or 16 or 17 or 18 or 19 or 20 or 21 or 22 or 23 or 24 or 25 or 26 or 27 or 28 or 29 or 30 or 31 or 32 or 33 or 34 or 35 or 36 or 37 or 38 or 39 or 40 or 41 or 42 or 43 or 44 or 45 or 46 or 47 or 48 or 49 or 50 or 51 or 52 or 53 or 54 or 55 or 56 or 57 or 58 or 59 or 60 or 61 or 62 or 63 or 64 | 467,496 |
| 66 | 9 and 14 and 65 | 369 |
| 67 | remove duplicates from 66 | **369** |

**SCOPUS SEARCH TERMS**

TITLE-ABS ( "schizophrenia spectrum" OR "other psychotic disorder*" OR psychiatric OR psycho* OR "bipolar disorder*" OR bipolar OR hypomania* OR mania* OR manic* OR schizoaffective OR schizo* OR psychotic* OR psychosis OR psychoses OR delusion* OR catatoni* OR hallucinat* OR hebephreni* OR oligophreni* OR paranoi* OR "schizoaffective psychosis" ) AND TITLE-ABS ( "weight reduc* program*" OR "weight loss" OR "body weight change*" OR "exercise*" OR "exercise movement technique*" OR "physical activit*" OR "sport*" OR "walk*" OR jog* OR swim* OR bicycl* OR danc* OR "weight training" OR "aerobic exercise*" OR "resistance train*" OR "body weight management" OR "behavio?r therap*" OR "behavior therap*" OR "cogniti* therap*" OR "lifestyle modification" OR "lifestyle intervention" ) AND TITLE-ABS ( ( "cost-benefit analysis" OR "cost analys?s" OR "cost-effectiveness" OR "cost*benefit*" OR "cost*utility" OR "cost*utilities" OR "cost*outcome*" OR "cost*consequence*" ) )

**Total: 500 document results**

**Section 3: List of included studies**

a Park, A. (2014). Exploring the Economic Implications of a Group-Based Lifestyle Intervention for Middle-Aged Adults with Chronic Schizophrenia and Co-Morbid Type 2 Diabetes [Article]. Journal of Diabetes & Metabolism, 5(5). <https://doi.org/10.4172/2155-6156.1000366>

Deenik, J., van Lieshout, C., van Driel, H. F., Frederix, G. W. J., Hendriksen, I. J. M., van Harten, P. N., & Tenback, D. E. (2022). Cost-Effectiveness of a Multidisciplinary Lifestyle-Enhancing Treatment for Inpatients With Severe Mental Illness: The MULTI Study V. Schizophrenia Bulletin Open, 3(1), sgac022. <https://doi.org/10.1093/schizbullopen/sgac022>

Heslin, M., Patel, A., Stahl, D., Gardner-Sood, P., Mushore, M., Smith, S., Greenwood, K., Onagbesan, O., O’Brien, C., Fung, C., Ohlsen, R., Hopkins, D., Lowe, P., Arbuthnot, M., Mutatsa, S., Todd, G., Kolliakou, A., Lally, J., Stubbs, B., … Gaughran, F. (2017). Randomised controlled trial to improve health and reduce substance use in established psychosis (IMPaCT): Cost-effectiveness of integrated psychosocial health promotion. BMC Psychiatry, 17(1). <https://doi.org/10.1186/s12888-017-1570-1>

Holt, R. I. G., Gossage-Worrall, R., Hind, D., Bradburn, M. J., McCrone, P., Morris, T., Edwardson, C., Barnard, K., Carey, M. E., Davies, M. J., Dickens, C. M., Doherty, Y., Etherington, A., French, P., Gaughran, F., Greenwood, K. E., Kalidindi, S., Khunti, K., Laugharne, R., … Wright, S. (2019). Structured lifestyle education for people with schizophrenia, schizoaffective disorder and first-episode psychosis (STEPWISE): Randomised controlled trial. British Journal of Psychiatry, 214(2), 63–73. <https://doi.org/10.1192/bjp.2018.167>

Janssen, E. M., Jerome, G. J., Dalcin, A. T., Gennusa, J. v., Goldsholl, S., Frick, K. D., Wang, N., Appel, L. J., & Daumit, G. L. (2017). A cost analysis of implementing a behavioral weight loss intervention in community mental health settings: Results from the ACHIEVE trial [Article]. Obesity (Silver Spring, Md.), 25(6), 1006–1013. <https://doi.org/10.1002/oby.21836>

Looijmans, A., Jörg, F., Bruggeman, R., Schoevers, R. A., Corpeleijn, E., Feenstra, T. L., & van Asselt, A. D. I. (2020). Cost-effectiveness and budget impact of a lifestyle intervention to improve cardiometabolic health in patients with severe mental illness. Global & Regional Health Technology Assessment, 7(1), 131–138. <https://doi.org/10.33393/grhta.2020.2027>

Meenan, R. T., Stumbo, S. P., Yarborough, M. T., Leo, M. C., Yarborough, B. J. H., & Green, C. A. (2016). An Economic Evaluation of a Weight Loss Intervention Program for People with Serious Mental Illnesses Taking Antipsychotic Medications. Administration and Policy in Mental Health and Mental Health Services Research, 43(4), 604–615. <https://doi.org/10.1007/s10488-015-0669-2>

Osborn, D., Burton, A., Hunter, R., Marston, L., Atkins, L., Barnes, T., Blackburn, R., Craig, T., Gilbert, H., Heinkel, S., Holt, R., King, M., Michie, S., Morris, R., Morris, S., Nazareth, I., Omar, R., Petersen, I., Peveler, R., … Walters, K. (2018). Clinical and cost-effectiveness of an intervention for reducing cholesterol and cardiovascular risk for people with severe mental illness in English primary care: a cluster randomised controlled trial. The Lancet Psychiatry, 5(2), 145–154. <https://doi.org/http://dx.doi.org/10.1016/S2215-0366%2818%2930007-5>

Verhaeghe, N., de Smedt, D., de Maeseneer, J., Maes, L., van Heeringen, C., & Annemans, L. (2014). Cost-effectiveness of health promotion targeting physical activity and healthy eating in mental health care [Article]. BMC Public Health, 14(1), 856–856. <https://doi.org/10.1186/1471-2458-14-856>

**Section 4: Quality assessment**

| **Quality Assessment Checklist** | Deenik et al. 2022 (44) | Heslin et al. 2017 (40) | Holt et al. 2019 (39) | Janssen et al. 2017 (42) | Looijmans et al. 2020 (43) | Meenan et al. 2016 (41) | Osborn et al. 2018 (13) | Park 2014 (12) | Verhaeghe et al. 2014 (45) |
| --- | --- | --- | --- | --- | --- | --- | --- | --- | --- |
| **1. Was a well-defined question posed in answerable form?** | Yes | Yes | Yes | Yes | Yes | Yes | Yes | Yes | Yes |
| Did the study examine both costs and effects of the service(s) or programme(s) over an appropriate time horizon? | Partly | Partly | Partly | Partly | Partly | Partly | Partly | Partly | Yes |
| Did the study involve a comparison of alternatives? | Yes | Yes | Yes | Yes | Yes | Yes | Yes | Yes | Yes |
| Was a perspective for the analysis stated and was the study placed in any particular decision-making context? | Yes | Yes | Yes | Yes | Yes | Yes | Yes | Yes | Yes |
| Were the patient population and any relevant subgroups adequately defined? | Yes | Yes | Yes | Yes | Yes | Yes | Yes | Yes | Yes |
| **2. Was a comprehensive description of the competing alternatives given? (i.e. can you tell who did what to whom, where, and how often?)** | Yes | Yes | Yes | Yes | Yes | Partly | Yes | Yes | Yes |
| Were any relevant alternatives omitted? | Can’t tell | No | No | Can’t tell | No | No | Can’t tell | No | Can’t tell |
| Was (should) a ‘do nothing’ alternative (be) considered? | N/A | N/A | No | N/A | n/a | n/a | n/a | N/A | n/a |
| Were relevant alternatives identified for the patient subgroups? | No | Yes | No | Yes | Yes | Yes | Yes | Yes | Yes |
| **3. Was the effectiveness of the programmes or services established?** | Yes | Yes | Yes | Yes | Yes | Yes | No | Partly | Yes |
| Was this done through a randomized controlled clinical trial? If so, did the trial protocol reflect what would happen in regular practice? | No | Yes | Yes | Partly | Yes | Yes | Yes | No | Yes |
| Were effectiveness data collected and summarized through a systematic overview of clinical studies? If so, were the search strategy and rules for inclusion or exclusion outlined? Was (should) a ‘do nothing’ alternative (be) considered? Were relevant alternatives identified for the patient subgroups? | N/A | N/A | No | N/A | N/A | N/A | N/A | Partly | No |
| Were observational data or assumptions used to establish effectiveness? If so, were any potential biases recognized? | Yes | No | No | No | No | No | No | No | No |
| **4. Were all the important and relevant costs and consequences for each alternative identified?** | No | Yes | Yes | Yes | Yes | Partly | Yes | Yes | Partly |
| Was the range wide enough for the research question at hand? | Yes | Yes | Yes | Can’t tell | Can’t tell | Yes | Yes | Yes | Can’t tell |
| Did it cover all relevant perspectives? (Possible perspectives include those of patients and third-party payers; other perspectivess may also be relevant depending on the particular analysis.) | Partly | Yes | No | Yes | Yes | Yes | Partly | Yes | Partly |
| Were capital costs, as well as operating costs, included? | Yes | Partly | Yes | Yes | Yes | Yes | Partly | Partly | No |
| **5. Were costs and consequences measured accurately in appropriate physical units prior to valuation (e.g. hours of nursing time, number of physician visits, lost work-days, gained life-years)?** | Yes | Yes | Yes | Yes | Yes | Yes | Yes | Yes | Yes |
| Were the sources of resource utilization described and justified? | Yes | Yes | Yes | Yes | Yes | Yes | Yes | Yes | Yes |
| Were any of the identified items omitted from measurement? If so, does this mean that they carried no weight in the subsequent analysis? | No | No | Yes | Can’t tell | No | No | No | No | No |
| Were there any special circumstances (e.g. joint use of resources) that made measurement difficult? Were these circumstances handled appropriately? | No | No | No | No | Can’t tell | Can’t tell | No | No | Can’t tell |
| **6. Were costs and consequences valued credibly?** | Yes | Yes | Yes | Partly | Yes | Yes | Yes | Yes | Yes |
| Were the sources of all values clearly identified? (Possible sources include market values, patient or client preferences and views, policymakers’ views, and health professionals’ judgements.) | Yes | Yes | Yes | Yes | Yes | Yes | Yes | Yes | Yes |
| Were market values employed for changes involving resources gained or depleted? | Yes | Yes | Yes | Yes | Yes | Yes | Yes | Yes | Yes |
| Where market values were absent (e.g. volunteer labour), or market values did not reflect actual values (e.g. clinic space donated at a reduced rate), were adjustments made to approximate market values? | Yes | N/A | Yes | Yes | N/A | Yes | N/A | N/A | N/A |
| Was the valuation of consequences appropriate for the question posed (i.e. has the appropriate type or types of analysis— cost-effectiveness, cost– benefit— been selected)? | Yes | Yes | Yes | Partly | Yes | Yes | Yes | Yes | Yes |
| **7. Were costs and consequences adjusted for differential timing?** | No | Yes | N/A | No | N/A | Can’t tell | N/A | N/A | Yes |
| Were costs and consequences that occur in the future ‘discounted’ to their present values? | No | Yes | No | No | N/A | no | N/A | N/A | Yes |
| Was any justification given for the discount rate(s) used? Was an incremental analysis of costs and consequences of alternatives performed? | No | Yes | No | N/A | N/A | no | N/A | N/A | Yes |
| **8. Was an incremental analysis of costs and consequences of alternatives performed?** | Yes | No | Yes | N/A | Yes | Yes | Yes | Yes | Yes |
| Were the additional (incremental) costs generated by one alternative over another compared to the additional effects, benefits, or utilities generated? | Yes | No | Yes | N/A | Yes | Yes | Partly | Yes | Yes |
| **9. Was uncertainty in the estimates of costs and consequences adequately characterized?** | Yes | Yes | Yes | Yes | Partly | Yes | Yes | Yes | Yes |
| If patient-level data on costs or consequences were available, were appropriate statistical analyses performed? | Yes | N/A | Yes | Yes | Yes | Yes | Yes | N/A | Yes |
| If a sensitivity analysis was employed, was justification provided for the form(s) of sensitivity analysis employed and the ranges or distributions of values (for key study parameters)? | Yes | Yes | No | Partly | No | no | N/A | Yes | Yes |
| Were the conclusions of the study sensitive to the uncertainty in the results, as quantified by the statistical and/or sensitivity analysis? | Yes | Yes | No | Yes | Yes | Yes | Yes | Yes | Yes |
| Was heterogeneity in the patient population recognized, for example by presenting study results for relevant subgroups? | Yes | Partly | No | Yes | Yes | Yes | Yes | N/A | Yes |
| **10. Did the presentation and discussion of study results include all issues of concern to users?** | Yes | Yes | No | Partly | Yes | Yes | Yes | Yes | Yes |
| Were the conclusions of the analysis based on some overall index or ratio of costs to consequences (e.g. cost-effectiveness ratio)? If so, was the index interpreted intelligently or in a mechanistic fashion? | Yes | Yes | No | No | Yes | Yes | Yes | Yes | Yes |
| Were the results compared with those of others who have investigated the same question? If so, were allowances made for potential differences in study methodology? | Yes | Yes | Yes | Yes | Yes | Yes | Yes | Yes | Yes |
| Did the study discuss the generalizability of the results to other settings and patient/client groups? | Yes | No | No | Yes | Yes | no | Yes | Yes | Yes |
| Did the study allude to, or take account of, other important factors in the choice or decision under consideration (e.g. distribution of costs and consequences, or relevant ethical issues)? | Yes | Yes | Yes | Partly | Yes | Yes | Yes | Yes | Yes |
| Did the study discuss issues of implementation, such as the feasibility of adopting the ‘preferred’ programme given existing financial or other constraints, and whether any freed resources could be redeployed to other worthwhile programmes? | No | Yes | No | Yes | Yes | Yes | No | Yes | Yes |
| Were the implications of uncertainty for decision-making, including the need for future research, explored? | Yes | Yes | Yes | Yes | Yes | Partly | Yes | Yes | Yes |

**Section 5: Grade table**

**Author(s):** Huajie Jin1*, Oluwafunso Kolawole1*, Zhengwei Wang1

**Question:** Physical Activity as Weight management intervention compared to Usual care in Schizophrenia and Bipolar Disorder as a cost-effective intervention

**Setting:** Primary and Secondary Care

**Bibliography:**

| **Certainty assessment** | | | | | | | **№ of patients** | | **Effect** | | **Certainty** | **Importance** |
| --- | --- | --- | --- | --- | --- | --- | --- | --- | --- | --- | --- | --- |
| **№ of studies** | **Study design** | **Risk of bias** | **Inconsistency** | **Indirectness** | **Imprecision** | **Other considerations** | **Physical Activity as Weight management intervention** | **Usual care** | **Relative (95% CI)** | **Absolute (95% CI)** |  |  |
| **Total Cost per patient (follow-up: mean 16 months)** | | | | | | | | | | | | |
| 2 | randomised trials | not serious | serious^a^ | not serious | not serious | none | 299/618 (48.4%) | 319/618 (51.6%) | not pooled | see comment | ⨁⨁⨁◯ Moderate | IMPORTANT |
| **ICER/QALY gained (follow-up: median 10.5 months)** | | | | | | | | | | | | |
| 3 | randomised trials | not serious | serious^b^ | not serious | not serious | none | 568/1015 (56.0%) | 447/1015 (44.0%) | not pooled | see comment | ⨁⨁⨁◯ Moderate | IMPORTANT |
| **ICER/clinical outcome (follow-up: median 18 months)** | | | | | | | | | | | | |
| 2 | randomised trials | not serious | not serious | not serious | not serious | publication bias strongly suspected^c^ | 114/208 (54.8%) | 94/208 (45.2%) | not pooled | see comment | ⨁⨁⨁◯ Moderate | IMPORTANT |
| **ICER/clinical outcome (follow-up: 18 years)** | | | | | | | | | | | | |
| 1 | observational studies | serious^d^ | not serious | not serious | serious^e^ | all plausible residual confounding would reduce the demonstrated effect dose response gradient | 65/114 (57.0%) | 49/114 (43.0%) | not pooled | see comment | ⨁⨁◯◯ Low | IMPORTANT |

**CI:** confidence interval

#### Explanations

a. Results were not similar across studies. Janssen et al suggest that the intervention is cost-effective at a higher cost, while Osborn et suggest that the intervention has a lower mean cost per patient, but also less health benefit

b. Results were not similar across studies. Heslin et al - no evidence of the additional quality of life/clinical benefit, there is also no evidence of cost-effectiveness. However, Verhaeghe et al, found that the intervention was cost-effective in men but not in women

c. Meenan et al, omitted information about the number of participants in the intervention group and control group separately

d. Deenik et al – Attition bias due to loss to follow up. 112 participants were included, 11 dropped out, no information about the number lost to follow-up

e. Deenik et al – although between Q1 2014 & Q3 2015, a 1kg decrease in weight saved 25 Euros per quarter year. Without outliers, 53euros were saved, ICER: –2288euros/QALY gained. Without outliers, -6564euros/QALY gained, there is wide 95%CI for the calculated delta change in costs between groups
